# Faith-Based Lifestyle Intervention for Diabetes Prevention Among Adults in Bangladesh: A Cluster Randomized Clinical Trial

**DOI:** 10.1001/jamanetworkopen.2025.38101

**Published:** 2025-10-20

**Authors:** Bishwajit Bhowmik, Tasnima Siddiquee, Sanjida Binte Munir, Hafiza Nasrin, Tanjimul Islam, Tamanna Afroz, Liya Quamrun Nesa, Shamim Khan, Shamima Akhtar, Farsana Rahman Khan, Tareen Ahmed, Faria Afsana, Faruque Pathan, Mohammad Feroz Amin, Sharif Mahmood, Rahat Iqbal Chowdhury, Nurul Islam, Rie Ozaki, Syed Zakir Hossain, Mohammad Robed Amin, Abul Kalam Azad Khan

**Affiliations:** 1 Centre for Global Health Research, Diabetic Association of Bangladesh, Dhaka, Bangladesh; 2 Department of Endocrinology, Bangladesh Institute of Research and Rehabilitation in Diabetes, Endocrine, and Metabolic Disorders, Dhaka, Bangladesh; 3 Department of Mathematics, University of Central Arkansas, Conway; 4 Noncommunicable Disease Control, Directorate General of Health Services, Dhaka, Bangladesh; 5 Japan International Cooperation Agency, Dhaka, Bangladesh

## Abstract

**Importance:**

The incidence of type 2 diabetes (T2D) is increasing in low- and middle-income countries. Effective, culturally tailored interventions are needed for diabetes prevention.

**Objective:**

To evaluate the effectiveness of a 12-month, mosque-based, faith-integrated lifestyle intervention in reducing T2D incidence among adults with prediabetes in rural Bangladesh.

**Design, Setting, and Participants:**

This parallel-group, cluster randomized clinical trial (RCT) was conducted from April 2022 to April 2023 across 8 rural mosque clusters in 5 districts of Bangladesh, with a 12-month follow-up. Adults (aged 25-65 years) were screened using a validated diabetes risk score. Individuals with high risk scores underwent oral glucose tolerance testing, and those with prediabetes (fasting blood glucose of 6.1-6.9 mmol/L, 2-hour blood glucose of 7.8-11.0 mmol/L [to convert to milligrams per deciliter, multiply by 18], or both, according to World Health Organization criteria) were included in this trial. Participants were randomized (1:1) by mosque cluster to the intervention or the control. After baseline assessments, participants who completed the 12-month follow-up were included in the intention-to-treat analysis.

**Intervention:**

The intervention group attended monthly mosque-based sessions integrating Islamic teachings with structured guidance on diet, physical activity, and behavior modification delivered by imams and trained female assistants. The control group received usual care (standard health advice including a health leaflet and referral to local health services) at enrollment.

**Main Outcomes and Measures:**

The primary outcome was the 12-month cumulative incidence of T2D. Secondary outcomes included changes in weight, body mass index (BMI), glycemic markers, lipid profiles, blood pressure, diabetes-related knowledge, physical activity, and quality of life.

**Results:**

Of the 799 participants (mean [SD] age, 46.2 [11.6] years; 424 women [53.1%]) randomized, 641 completed the 12-month follow-up and were included in the intention-to-treat analysis: 341 (n = 4 mosques) in the intervention group and 300 (n = 4 mosques) in the control group. The intervention group had a significantly lower 12-month cumulative T2D incidence (9.8% [95% CI, 7.1%-13.5%]) vs the control group (17.1% [95% CI, 13.4%-21.6%]), with an absolute risk reduction of 7.3 percentage points (95% CI, 5.2-10.6 percentage points), a relative risk reduction of 42.5% (95% CI, 15.0%-70.0%), and a number needed to treat of 14 (95% CI, 9-39). In Cox proportional hazards regression adjusted for clustering, the hazard ratio was 0.75 (95% CI, 0.60-0.95; *P* = .02), corresponding to a 25.0% reduction in diabetes risk. Secondary outcomes including weight, BMI, fasting glucose, 2-hour blood glucose, hemoglobin A_1c_, lipid profiles, diabetes-related knowledge, physical activity, and quality of life also improved significantly more in the intervention group compared with the control group.

**Conclusions and Relevance:**

In this cluster RCT involving adults with prediabetes, a culturally adapted, mosque-based lifestyle intervention significantly reduced T2D incidence over 12 months in rural Bangladesh. These results demonstrate the potential for scalable and cost-effective diabetes prevention programs in low-resource, Muslim-majority communities.

**Trial Registration:**

ISRCTN Registry Identifier: ISRCTN91564707

## Key Points

**Question:**

Can a culturally tailored, faith-based lifestyle intervention reduce the incidence of type 2 diabetes (T2D) among adults with prediabetes in rural Bangladesh?

**Findings:**

In this cluster randomized clinical trial involving 799 adults with prediabetes, participants who received a 12-month, mosque-based, faith-integrated lifestyle intervention had a 43% relative risk reduction in T2D incidence compared with the control group.

**Meaning:**

This trial found that a faith-based intervention decreased new-onset T2D incidence at 12 months, suggesting it may be an effective and scalable strategy for T2D prevention in low-resource, Muslim-majority communities.

## Teaser

This cluster randomized clinical trial evaluates whether a 12-month, mosque-based, faith-integrated lifestyle intervention decreases the incidence of new-onset type 2 diabetes among adults with prediabetes in rural Bangladesh.

## Introduction

Type 2 diabetes (T2D) is an escalating global health concern, with more than 75% of cases occurring in low- and middle-income countries (LMICs). Bangladesh currently ranks eighth in T2D incidence worldwide, with more than 13.9 million individuals affected, which is projected to increase sharply by 2050.^1^ Rapid urbanization, sedentary lifestyles, and widespread adoption of energy-dense diets have contributed to rising obesity, hypertension, and dyslipidemia—key risk factors for diabetes.^2,3^ These trends place a growing economic burden on already constrained health care systems, particularly in rural areas.^4,5^

Landmark trials, such as the Diabetes Prevention Program (DPP), the Finnish Diabetes Prevention Study (DPS), and the Da Qing Study, demonstrated that intensive lifestyle interventions can reduce diabetes incidence by 42% to 58%.^6,7,8^ However, translating these interventions to resource-limited LMIC settings remains a major challenge due to limitations in infrastructure, funding, and sociocultural adaptation.^9,10^ Local adaptations in India and Bangladesh have shown promise but often struggle with long-term sustainability and scalability.^11,12^

Faith-based interventions offer a culturally resonant and potentially scalable strategy in contexts like Bangladesh, where more than 90% of the population is Muslim and mosques serve as central community institutions. Islamic principles emphasizing moderation, healthy eating, and physical activity align closely with diabetes prevention goals.^13,14^ Successful faith-based models include the Body and Soul program in African American churches in the US and mosque-based health promotion efforts in Canada.^13,14^ In Bangladesh, a previous study of the Diabetes Prevention Through Religious Leaders initiative demonstrated its feasibility and community engagement, although rigorous evidence on clinical effectiveness remains limited.^15^

For this trial, a 12-month intervention and follow-up period was selected based on evidence from prior randomized clinical trials (RCTs) showing significant benefits within this time frame, as well as logistical feasibility in rural Bangladesh to assess diabetes progression and early behavioral sustainability through faith-integrated counseling.^13,14,16,17,18,19,20^ This RCT aimed to evaluate the effectiveness of a culturally tailored, mosque-based lifestyle intervention in reducing T2D incidence and improving cardiometabolic outcomes among adults with prediabetes in rural Bangladesh, addressing a critical evidence gap in scalable prevention models for LMICs.

## Methods

### Trial Design and Oversight

This 12-month, parallel-group, cluster RCT was conducted from April 2022 to April 2023 across 8 mosque clusters in 5 rural districts of Bangladesh (Manikganj, Narsingdi, Munshiganj, Dhaka, and Tangail), with each cluster defined at the mosque level. The trial followed the Consolidated Standards of Reporting Trials (CONSORT) reporting guideline for cluster designs. Ethical approval was granted by the Diabetic Association of Bangladesh, and the study complied with the Declaration of Helsinki.^21^ All participants gave written informed consent after receiving study information in their native language. The process was conducted independently by trained research staff outside of formal religious services. Participation was voluntary and could be withdrawn at any time without consequence. Oversight was provided by an independent steering committee and a data monitoring and ethics committee. The full trial protocol and the statistical analysis plan are available in Supplement 1.

### Participants

Adults aged 25 to 65 years were recruited from mosque congregations in 5 rural districts of Bangladesh via community announcements and health camps. Of the 12 000 individuals approached, 10 224 met preliminary eligibility criteria, including permanent residency, consent capacity, and availability for 12-month follow-up.

Individuals with a diabetes risk score of 9 or greater (considered high risk) were identified using a validated mobile application–based tool.^22^ Of the 5127 individuals who completed an oral glucose tolerance test (OGTT), 1914 had OGTT results meeting the 2006 World Health Organization (WHO) criteria for prediabetes (fasting blood glucose [FBG] of 6.1-6.9 mmol/L, 2-hour blood glucose [BG] of 7.8-11.0 mmol/L [to convert to milligrams per deciliter, multiply by 18], or both).^23^ Of these, 824 eligible and consenting individuals were enrolled across 8 mosque clusters and randomized 1:1 to intervention (n = 396) or control (n = 403) groups stratified by cluster. After excluding 25 individuals lost before baseline, 799 participants were included in the final analysis.

Exclusion criteria included prior T2D diagnosis, pregnancy, lactation, serious illness, or planned transfer. Only 1 participant per household was enrolled to limit intrafamily influence. No imams or female facilitators were enrolled in either group.

### Timing of Outcome Assessments

OGTTs were conducted at baseline, 4 months, and 12 months to assess glycemic status and classify individuals by WHO 2006 diagnostic criteria.^23^ For enrolled participants with prediabetes, follow-up OGTTs evaluated glycemic progression or regression. Secondary outcomes—including anthropometric, clinical, biochemical, behavioral, and quality-of-life measures—were assessed at the same time points to capture short-term and sustained effects of the intervention.

### Randomization and Blinding

Eight geographically distinct mosque clusters were randomized 1:1 to either the intervention group or the control group using a computer-generated block randomization sequence (block size of 2) stratified by district. The intervention group included mosques in Shibaloy, Monohardi, Raipura, and Dhanbari; the control group included mosques in Singair, Keraniganj, Sreenagar, and Sakhipur. Randomization was conducted centrally by an independent statistician using sealed opaque envelopes. Participant enrollment was facilitated by trained field supervisors, using the national voter list to identify eligible individuals from mosque catchment areas. Recruitment and screening were conducted at mosque sites.

Due to the behavioral nature of the intervention, neither participants nor facilitators (imams and female assistants) were blinded. However, outcome assessors and data analysts were blinded to randomization to reduce detection and analytical bias. Implementers were uniquely assigned to their respective mosque clusters and were not shared or rotated between groups, ensuring fidelity to intervention delivery.

To minimize contamination, mosque clusters were separated by 50 to 60 miles within districts and 100 to 300 miles across districts (to convert miles to kilometers, multiply by 1.6). Randomization was stratified by district to account for regional variability. These design elements—including geographic separation and fixed implementer assignment—enhanced internal validity while supporting pragmatic implementation in community settings.

### Intervention

Participants in the intervention group received a 12-month, mosque-based lifestyle program combining faith-based messaging with dietary and physical activity counseling. Monthly 2-hour group sessions were held at mosque sites, beginning with 15-minute sermons by trained imams (for men) or female assistants (for women), emphasizing Islamic principles of moderation, health responsibility, and disease prevention.

Each session included 15 minutes of dietary counseling based on WHO and national guidelines,^24^ focusing on reducing intake of rice, salt, oil, sugar, and sugary drinks while promoting intake of fruits, vegetables, and whole grains. Culturally adapted plate models were used by female assistants trained by certified dietitians. A 30-minute physical activity component followed, with demonstrations and encouragement for daily walking (morning walks with imams for men and afternoon walks with female assistants for women). Participants were encouraged to meet WHO-recommended physical activity levels (≥30 minutes per day).

Adherence was documented using individual guidebooks maintained by trained local volunteers, recording sermon attendance, walking participation, dietary changes, and follow-up visits. Monthly weight and blood pressure were also monitored. Logs were reviewed monthly by field supervisors, and adherence was reinforced through checklist-based audits, refresher trainings, and small incentives for imams and female assistants.

The control group received standard care, including a health leaflet and referral to local health services. No structured sessions were delivered.

### Outcome Measures

The primary outcome was 12-month cumulative incidence of T2D, defined by WHO 2006 criteria (FBG ≥7.0 mmol/L, 2-hour BG ≥11.1 mmol/L, or both),^23^ assessed via OGTT at baseline, 4 months, and 12 months by trained staff on-site. No outcome data were extracted from medical records.

Secondary outcomes included changes from baseline to 12 months in a range of anthropometric, clinical, behavioral, and psychosocial measures. Anthropometric parameters assessed were body weight, body mass index (BMI), waist and hip circumference, and waist-to-hip ratio. Glycemic control was evaluated using FBG, 2-hour BG, and glycated hemoglobin (HbA_1c_). Lipid profiles included total cholesterol, high-density lipoprotein cholesterol (HDL-C), low-density lipoprotein cholesterol (LDL-C), and triglyceride levels. Measurements were recorded for both systolic and diastolic blood pressure readings. Health-related quality of life was assessed using the EuroQol 5-Dimension 5-Level (EQ-5D-5L) instrument and the EuroQol visual analog scale (EQ-VAS). In addition, diabetes-related knowledge, dietary intake, and physical activity were measured using structured, validated questionnaires.

Capillary blood samples (FBG and 2-hour BG) were analyzed using point-of-care devices for glucose (Alere G1; Allmed), HbA_1c_ (Affinion 2; Abbott), and lipids (Cholestech LDX analyzer; Abbott). All outcome assessors were blinded to group assignment. Data quality was ensured through standardized procedures, duplicate measurements, monthly calibration, and external validation of 10% of samples. Additional cardiometabolic assessments, including electrocardiography and fundoscopy, were performed at each time point.

### Sample Size Calculation

The sample size for this cluster RCT was calculated to detect a 50% relative risk reduction (RRR) in the cumulative incidence of T2D in the intervention group compared with the control group (from 18% to 9%).^25^ The calculation assumed 80% power, a 2-sided α = .05, and an intraclass correlation coefficient (ICC) of 0.05 to account for clustering within mosques.

The initial estimate, adjusted for a design effect of 6.1, required 206 participants per group (before accounting for attrition). To address a potential 50% loss to follow-up, this was inflated to 412 participants per group (n = 824 total), distributed across 8 mosque clusters (approximately 103 participants per cluster).

### Statistical Analysis

All analyses adhered to the intention-to-treat principle. Baseline characteristics were compared using *t* tests and χ^2^ tests, with results reported as means (SDs) or proportions.

The primary outcome (12-month cumulative incidence of T2D) was assessed using a Cox proportional hazards regression model with shared frailty to account for mosque-level clustering. Group differences were visualized via Kaplan-Meier curves and log-rank tests; proportional hazards assumptions were evaluated using Schoenfeld residuals and log-minus-log plots. Hazard ratios (HRs) with 95% CIs, absolute risk reduction (ARR), RRR, and number needed to treat (NNT) were calculated.

Time-to-event differences were further examined using restricted mean survival time (RMST) analysis, with bootstrapped 95% CIs estimating average diabetes-free survival. Diabetes incidence rates are expressed per 1000 person-years, adjusted for attrition. The ICC for the primary outcome was 0.04 (95% CI, 0.02-0.09).

Continuous secondary outcomes (eg, BMI, glucose, and lipid levels) were analyzed using linear mixed-effects models. Binary outcomes (eg, normoglycemia and physical activity) used generalized estimating equations (GEEs), incorporating group, time, and group-by-time interaction terms.

Model diagnostics included C statistics for the Cox regression model (C statistic = 0.74), Q-Q plots, residual plots, and the quasi-likelihood information criterion for GEEs. Sensitivity analyses employed Poisson regression, discrete-time survival models, and interval-censored Cox regression models (eTable 5 in Supplement 2). Missing data were addressed using complete-case analysis, multiple imputation by chained equations, and worst-case imputation scenarios for dropouts.

Two-sided *P* < .05 was considered statistically significant. All analyses were conducted in Stata, version 17.0 (StataCorp).

## Results

### Participant Flow and Retention

**Figure 1.**
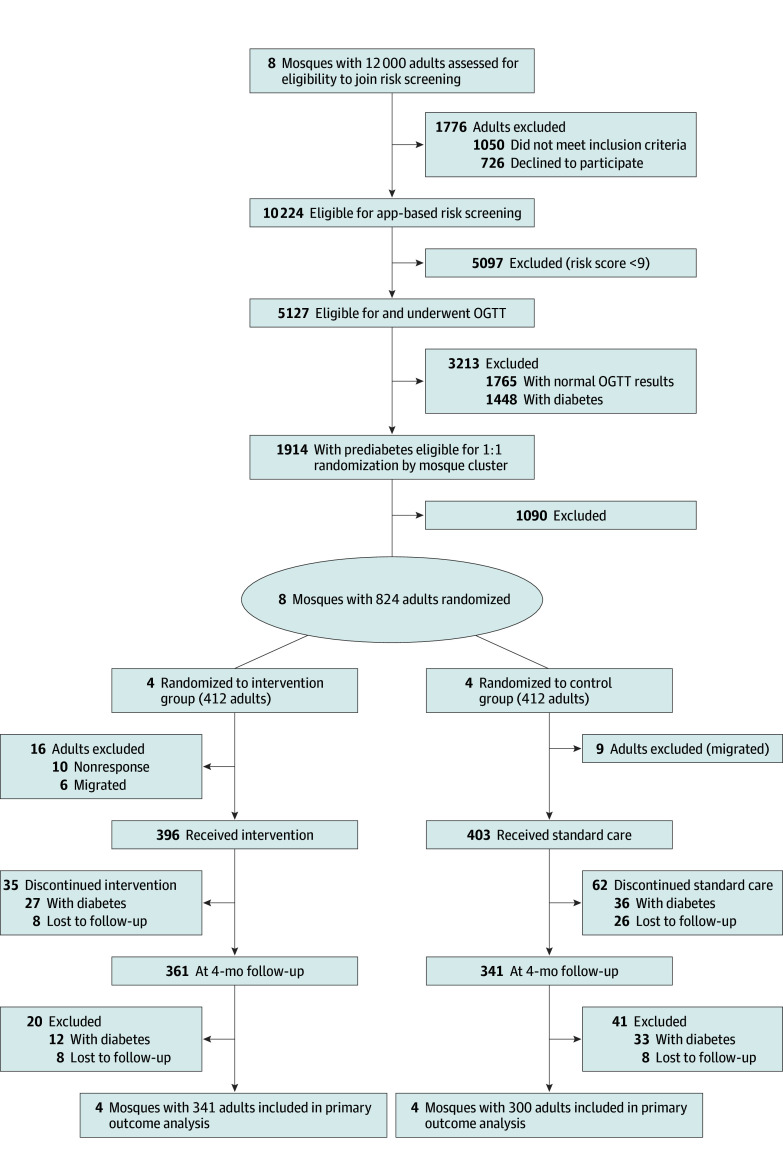
CONSORT Flow Diagram OGTT indicates oral glucose tolerance test.

A total of 799 participants (n = 8 mosques) were randomized to the intervention group (n = 396 [4 mosques]) or control group (n = 403 [4 mosques]). Their mean (SD) age was 46.2 (11.6) years; 424 (53.1%) were women and 375 (46.9%) were men. Baseline characteristics were comparable between groups (Table 1). A total of 341 individuals (n = 4 mosques) in the intervention group and 300 (n = 4 mosques) in the control group completed the 12-month follow-up and were included in the primary intention-to-treat analysis. Reasons for exclusion and loss to follow-up are detailed in Figure 1. Retention was higher in the intervention group (341 of 396 participants [86.1%]) than in the control group (300 of 403 participants [74.4%]). No significant differences were observed in sociodemographic, clinical, or behavioral variables. Cluster-level comparisons and dropout analyses (eTables 1 and 2 in Supplement 2) indicated successful randomization and minimal attrition bias.

**Table 1.** Baseline Characteristics of Study Participants^a^

Variable	All participants (N = 799)	Control group (n = 403)	Intervention group (n = 396)
Age, y			
Mean (SD)	46.2 (11.6)	46.8 (12.2)	45.6 (10.9)
Group			
<40	239 (29.9)	129 (32.0)	110 (27.8)
≥40	560 (70.1)	274 (68.0)	286 (72.2)
Sex			
Female	424 (53.1)	205 (50.9)	219 (55.3)
Male	375 (46.9)	198 (49.1)	177 (44.7)
Formal education			
No	333 (41.7)	156 (38.7)	177 (44.7)
Yes	466 (58.3)	247 (61.3)	219 (55.3)
Occupation			
Homemaker	393 (49.2)	196 (48.6)	197 (49.7)
Other	406 (50.8)	207 (51.4)	199 (50.3)
Monthly expenditures, mean (SD), Bangladeshi taka	17 497 (1254)	18 230 (1346)	16 751 (1149)
Earning capacity			
Earner	374 (46.8)	194 (48.1)	180 (45.5)
Dependent	425 (53.2)	209 (51.9)	216 (54.5)
Physical activity			
Inactive	455 (56.9)	218 (54.1)	237 (59.8)
Active	344 (43.1)	185 (45.9)	159 (40.2)
Smoker	215 (26.9)	99 (24.6)	106 (26.8)
Dietary intake, mean (SD), kcal/d	1329.6 (310.9)	1336.5 (319.7)	1322.7 (301.9)
Family history of diabetes (positive)	209 (26.9)	113 (28.0)	102 (25.8)
Diabetes knowledge (some)	290 (37.2)	150 (37.9)	140 (36.5)
Health-related quality of life assessment score, mean (SD)			
EQ-5D-5L	3.01 (1.76)	3.01 (1.76)	3.00 (1.76)
EQ-VAS	64.4 (14.3)	65.2 (15.6)	63.6 (14.8)
Weight, mean (SD), kg	62.3 (12.0)	62.4 (11.8)	62.3 (12.3)
BMI, mean (SD)	25.2 (4.2)	25.4 (4.2)	25.0 (4.2)
Waist circumference, mean (SD), cm	88.8 (12.0)	88.9 (12.0)	88.7 (11.9)
Waist-to-hip ratio, mean (SD)	0.94 (0.08)	0.94 (0.08)	0.93 (0.08)
Blood pressure, mean (SD), mm Hg			
Systolic	127.7 (19.7)	128.6 (19.6)	126.7 (19.8)
Diastolic	80.9 (11.2)	81.3 (10.7)	80.7 (11.7)
Blood glucose, mean (SD), mmol/L			
Fasting blood	6.1 (0.46)	6.1 (0.43)	6.1 (0.48)
2 h	7.9 (1.3)	7.9 (1.2)	7.9 (1.4)
Random capillary	7.6 (1.6)	7.6 (1.5)	7.5 (1.7)
HbA_1c_, mean (SD), %	5.8 (0.39)	5.8 (0.38)	5.8 (0.41)
Cholesterol level, mean (SD), mg/dL			
Total	182.1 (43.4)	183.2 (45.6)	180.9 (41.0)
HDL-C	37.6 (10.9)	37.6 (11.3)	37.5 (10.5)
LDL-C	112.5 (33.4)	114.1 (34.9)	110.8 (31.7)
Triglycerides, mean (SD), mg/dL	197.1 (113.7)	198.2 (116.6)	195.8 (110.8)
Urine albumin (positive)	100 (12.5)	49 (12.2)	51 (12.9)

Abbreviations: BMI, body mass index (calculated as weight in kilograms divided by height in meters squared); EQ-5D-5L, EuroQol 5-Dimension 5-Level; EQ-VAS, EuroQol visual analog scale; HbA_1c_, glycated hemoglobin; HDL-C, high-density lipoprotein cholesterol; LDL-C, low-density lipoprotein cholesterol.

SI conversion factors: To convert Bangladeshi taka to US $, multiply by 0.0082; glucose to milligrams per deciliter, multiply by 18; HbA1c to proportion of total hemoglobin, multiply by 0.01; total cholesterol, HDL-C, and LDL-C to millimoles per liter, multiply by 0.0259; triglycerides to millimoles per liter, multiply by 0.0113.

^a^ Unless indicated otherwise, values are presented as No. (%) of participants.

### Primary Outcome: T2D Incidence

**Figure 2.**
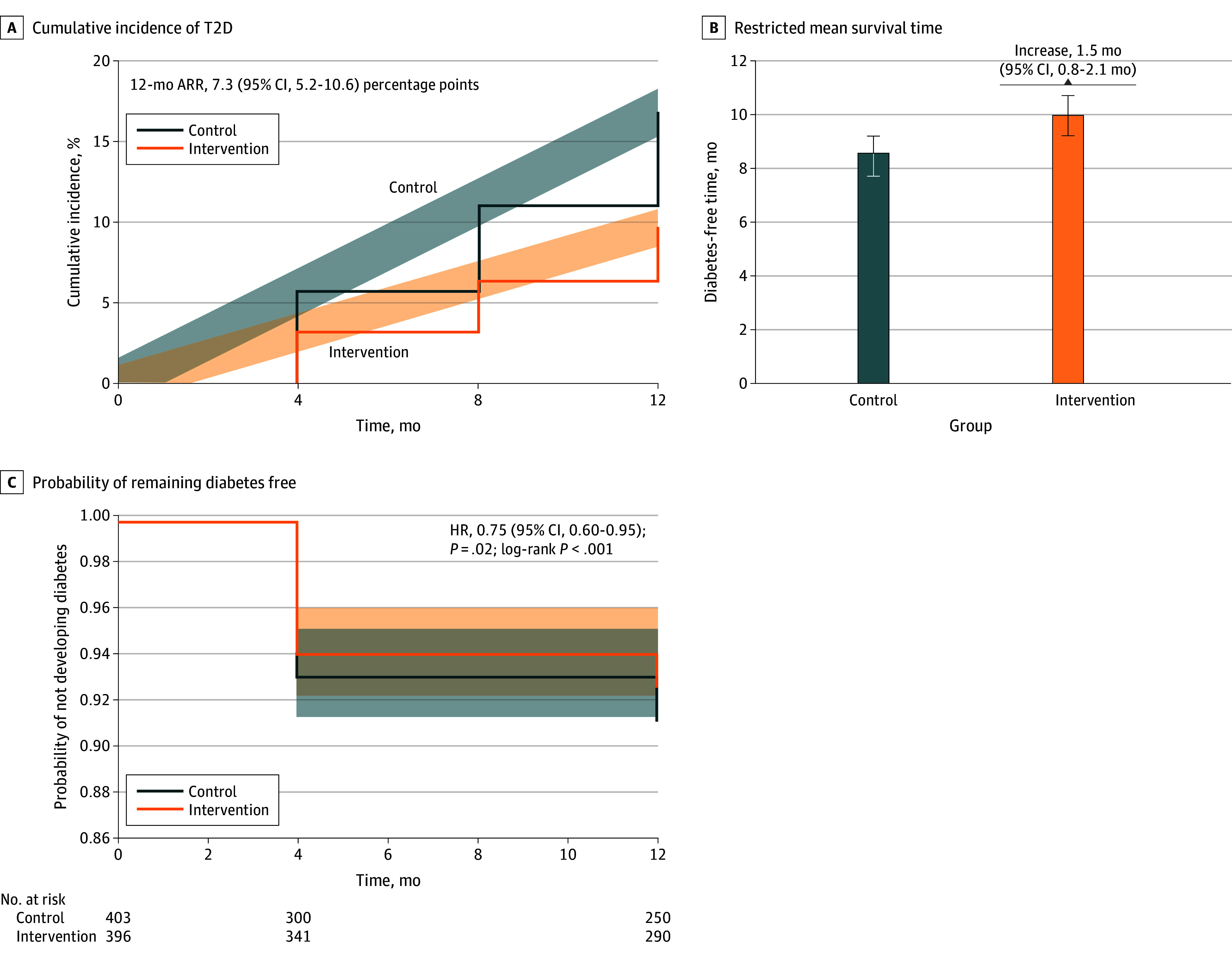
Effects of Faith-Based Lifestyle Intervention on Type 2 Diabetes (T2D) Incidence and Time to Diabetes Outcomes Over 12 Months A, Cumulative incidence of T2D. B, Restricted mean survival time (RMST). Bar plots compare average diabetes-free time (in months) between groups. C, Kaplan-Meier survival curves depict the probability of remaining free from diabetes over time.

At 12 months, the cumulative incidence of T2D was significantly lower in the intervention group (9.8% [95% CI, 7.1%-13.5%]) than in the control group (17.1% [95% CI, 13.4%-21.6%]) (Figure 2A), resulting in an ARR of 7.3 percentage points (95% CI, 5.2-10.6 percentage points; *P* = .002), an RRR of 42.5% (95% CI, 15.0%-70.0%), and a NNT of 14 (95% CI, 9-39) (eTable 3 in Supplement 2). Additionally, RMST analysis demonstrated that participants in the intervention group remained diabetes free for an average of 1.5 months longer than those in the control group (RMST: 10.0 vs 8.5 months [95% CI, 0.8-2.1 months]) (Figure 2B).

Time-to-event analysis using Kaplan-Meier survival curves (Figure 2C) showed greater diabetes-free survival in the intervention group (log-rank *P* < .001). Cox proportional hazards regression, adjusted for clustering, yielded an HR of 0.75 (95% CI, 0.60-0.95; *P* = .02), corresponding to a 25.0% reduction in diabetes risk.

### Secondary Outcomes: Cardiometabolic and Behavioral Changes

At 12 months, the intervention group showed significantly greater improvements in cardiometabolic outcomes than the control group (Table 2). Mean body weight decreased by 5.4 kg (95% CI, −6.1 to −4.7 kg) in the intervention group vs 1.1 kg (95% CI, −1.8 to −0.4 kg) in the control group (*P* < .001), with larger reductions in mean BMI (−1.4 [95% CI, −1.6 to −1.2] vs −0.2 [95% CI, −0.4 to 0]) and mean waist circumference (−6.0 cm [95% CI, −7.0 to −5.0 cm] vs −2.1 cm [95% CI, −3.0 to −1.2 cm]) (both *P* < .001). Glycemic markers also improved significantly in the intervention group compared with the control group: FBG decreased by 0.6 mmol/L (95% CI, −0.7 to −0.5 mmol/L), 2-hour BG by 1.5 mmol/L (95% CI, −1.8 to −1.2 mmol/L), and HbA_1c_ by 0.4% (95% CI, −0.5% to −0.3%) (to convert to proportion of total hemoglobin, multiply by 0.01) (all *P* < .001). Total cholesterol level decreased by 19.7 mg/dL (95% CI, −22.6 to −16.8 mg/dL), and HDL-C level increased by 0.5 mg/dL (95% CI, −0.7 to 1.7 mg/dL) (*P* = .003) (to convert total cholesterol and HDL-C to millimoles per liter, multiply by 0.0259). Diastolic blood pressure decreased by 2.9 mm Hg (95% CI, −4.0 to −1.8 mm Hg) in the intervention group vs 0.7 mm Hg (95% CI, −1.8 to 0.4 mm Hg) in the control group (*P* = .02).

**Table 2.** Changes in Anthropometric, Clinical, Biochemical, Behavioral, and Quality-of-Life Parameters Over 12 Months Between the Intervention and Control Groups

Outcome	Control group				Intervention group				Between groups	
	Mean (SD)			Change at 12 mo (95% CI)	Mean (SD)			Change at 12 mo (95% CI)	Difference at 12 mo (95% CI)	*P* value
	Baseline	4 mo	12 mo		Baseline	4 mo	12 mo			
Weight, kg	62.4 (11.8)	60.9 (11.2)	61.3 (11.3)	−1.1 (−1.8 to −0.4)^a^	62.3 (12.3)	57.3 (11.6)	56.9 (12.4)	−5.4 (−6.1 to −4.7)^b^	−4.3 (−5.3 to −3.3)	<.001
BMI	25.4 (4.2)	24.9 (3.9)	25.2 (4.0)	−0.2 (−0.4 to 0)^a^	25.0 (4.2)	23.7 (4.0)	23.6 (4.4)	−1.4 (−1.6 to −1.2)^b^	−1.2 (−1.5 to −0.9)	<.001
Waist circumference, cm	88.9 (12.0)	85.3 (10.5)	86.8 (10.9)	−2.1 (−3.0 to −1.2)^a^	88.7 (11.9)	83.6 (10.1)	82.7 (11.2)	−6.0 (−7.0 to −5.0)^b^	−3.9 (−5.3 to −2.5)	<.001
Hip circumference, cm	94.9 (11.1)	92.6 (8.5)	93.6 (8.7)	−1.3 (−1.8 to −0.8)^a^	95.1 (11.2)	91.1 (9.7)	90.6 (9.1)	−4.5 (−5.1 to −3.9)^b^	−3.2 (−4.0 to −2.4)	<.001
Waist-to-hip ratio	0.94 (0.08)	0.92 (0.08)	0.93 (0.07)	−0.01 (−0.02 to 0)^a^	0.93 (0.09)	0.92 (0.08)	0.92 (0.08)	−0.01 (−0.02 to 0)	0 (−0.01 to 0.01)	.34
Blood pressure, mm Hg										
Systolic	128.6 (19.6)	126.1 (21.9)	122.4 (17.9)	−6.2 (−8.0 to −4.4)^b^	126.7 (19.8)	120.0 (17.0)	117.7 (16.6)	−9.0 (−10.8 to −7.2)^b^	−2.8 (−5.4 to −0.2)	.03
Diastolic	81.3 (10.9)	80.4 (11.1)	80.6 (10.6)	−0.7 (−1.8 to 0.4)	80.7 (11.7)	78.1 (10.7)	77.8 (10.7)	−2.9 (−4.0 to −1.8)^b^	−2.2 (−3.8 to −0.6)	.007
Blood glucose, mmol/L										
Fasting	6.1 (0.43)	5.9 (0.78)	5.8 (0.76)	−0.3 (−0.4 to −0.2)^b^	6.1 (0.48)	5.7 (0.77)	5.5 (0.78)	−0.6 (−0.7 to −0.5)^b^	−0.3 (−0.5 to −0.1)	<.001
2 h	7.9 (1.2)	7.3 (1.9)	7.5 (2.0)	−0.4 (−0.7 to −0.1)^b^	7.9 (1.4)	6.8 (1.7)	6.2 (1.5)	−1.5 (−1.8 to −1.2)^b^	−1.1 (−1.5 to −0.7)	<.001
HbA_1c_, %	5.8 (0.38)	5.8 (0.51)	5.6 (0.47)	−0.2 (−0.3 to −0.1)^b^	5.8 (0.41)	5.7 (0.46)	5.4 (0.44)	−0.4 (−0.5 to −0.3)^b^	−0.2 (−0.3 to −0.1)	<.001
Cholesterol level, mg/dL										
Total	183.2 (45.5)	173.0 (39.4)	170.1 (38.6)	−13.1 (−16.0 to −10.2)^b^	180.9 (40.9)	167.4 (39.9)	161.2 (35.1)	−19.7 (−22.6 to −16.8)^b^	−6.6 (−10.8 to −2.4)	.002
HDL-C	37.6 (10.7)	33.4 (10.7)	35.0 (9.9)	−2.6 (−3.8 to −1.4)^b^	37.5 (10.5)	31.3 (11.4)	37.0 (8.8)	0.5 (−0.7 to 1.7)	3.1 (1.5-4.7)	.003
LDL-C	114.1 (34.9)	109.3 (30.9)	109.6 (29.4)	−4.5 (−7.1 to −1.9)^a^	110.8 (31.7)	104.4 (29.9)	103.2 (27.6)	−7.6 (−10.2 to −5.0)^b^	−3.1 (−6.7 to 0.5)	.09
Triglycerides, mg/dL	198.2 (116.6)	177.7 (104.9)	175.9 (87.3)	−22.3 (−30.1 to −14.5)^b^	195.8 (110.8)	182.4 (103.6)	169.7 (109.3)	−26.1 (−34.9 to −17.3)^b^	−3.8 (−15.6 to 8.0)	.53
Health-related quality of life assessment score										
EQ-5D-5L	3.01 (1.8)	3.30 (1.7)	2.91 (1.7)	−0.10 (−0.30 to 0.10)	3.00 (1.7)	2.88 (1.8)	3.33 (1.7)	0.33 (0.13-0.53)^a^	0.43 (0.15-0.71)	.003
EQ-VAS	65.2 (15.6)	67.3 (13.4)	66.8 (14.4)	1.6 (0.2-3.0)^a^	63.6 (14.8)	63.2 (14.0)	71.9 (13.9)	8.3 (6.9-9.7)^b^	6.7 (4.7-8.7)	<.001
Physically active, No. (%)	185 (45.9)	203 (53.3)	125 (56.6)	10.7 (4.9-16.5)^b^	159 (40.2)	181 (46.6)	214 (69.5)	29.3 (23.5-35.1)^b^	18.6 (11.2-26.0)	<.001
Diabetes knowledge, No. (%)	150 (37.9)	80 (22.2)	71 (22.3)	−15.6 (−20.8 to −10.4)^b^	140 (36.5)	106 (28.7)	219 (67.0)	30.5 (25.3-35.7)^b^	46.1 (39.7-52.5)	<.001

Abbreviations: BMI, body mass index (calculated as weight in kilograms divided by height in meters squared); EQ-5D-5L, EuroQol 5-Dimension 5-Level; EQ-VAS, EuroQol visual analog scale; HbA_1c_, glycated hemoglobin; HDL-C, high-density lipoprotein cholesterol; LDL-C, low-density lipoprotein cholesterol.

SI conversion factors: To convert glucose to milligrams per deciliter, multiply by 18; HbA1c to proportion of total hemoglobin, multiply by 0.01; total cholesterol, HDL-C, and LDL-C to millimoles per liter, multiply by 0.0259; triglycerides to millimoles per liter, multiply by 0.0113.

^a^ *P* < .05.

^b^ *P* < .001.

Quality-of-life indicators improved notably in the intervention group. EQ-5D-5L index scores increased by 0.33 points (95% CI, 0.13-0.53 points) in the intervention group, whereas they decreased by 0.10 points (95% CI, −0.30 to 0.10 points) in the control group (*P* = .005). Similarly, EQ-VAS scores rose by 8.3 points (95% CI, 6.9-9.7 points) in the intervention group vs 1.6 points (95% CI, 0.2-3.0 points) in the control group (*P* < .001). Behavioral outcomes also favored the intervention compared with the control: physical activity increased by 29.3% (95% CI, 23.5%-35.1%) vs 10.7% (95% CI, 4.9%-16.5%) (*P* = .04), and diabetes knowledge rose by 30.5% (95% CI, 25.3%-35.7%) in the control group compared with a 15.6% decrease (95% CI, −20.8% to −10.4%) in the control group (*P* < .001).

### Transitions in Glycemic Status

**Figure 3.**
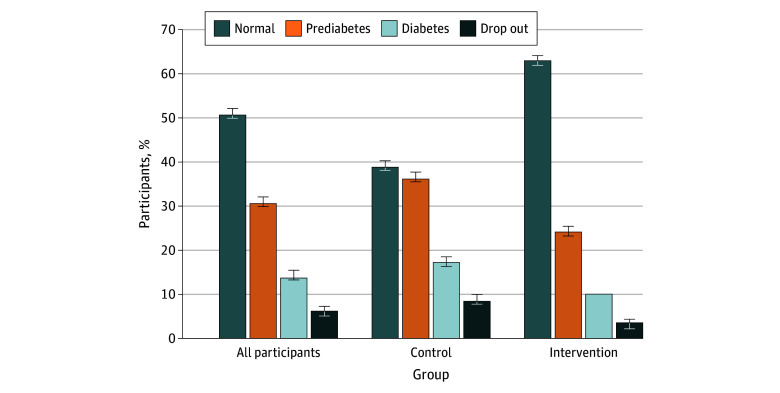
Change in Glycemic Status From Baseline to 12 Months in the Intervention and Control Groups Bars represent the proportion of participants in each glycemic category (normal, prediabetes, diabetes, or dropout) after 12 months. Percentages are grouped by study group (total population, control group, and intervention group). Error bars indicate 95% CIs. *P* < .001 for control vs intervention groups in all 4 glycemic categories.

At 12 months, 249 participants (62.9%) in the intervention group reverted to normoglycemia compared with 155 (38.5%) in the control group. Fewer participants in the intervention group progressed to diabetes or remained in a prediabetic state (Figure 3).

### Sensitivity Analyses

Multiple sensitivity analyses (complete-case, multiple imputation, worst-case, and as-observed scenarios) confirmed the robustness of the primary results (eTable 4 in Supplement 2). HRs for the primary outcome ranged from 0.74 (95% CI, 0.69-0.78) to 0.76 (95% CI, 0.70-0.79) across models. Improvements in glycemic indices remained consistent (adjusted changes, −1.2 to −0.9 units), indicating minimal impact from missing data or dropout bias.

## Discussion

This cluster RCT assessed the effectiveness of a culturally tailored, mosque-based lifestyle intervention in preventing T2D among adults with prediabetes in rural Bangladesh over a 12-month period. The results demonstrated a statistically significant reduction in the cumulative incidence of T2D in the intervention group compared with the control group, alongside favorable improvements in glycemic control, lipid profile, anthropometric measures, quality of life, and diabetes-related knowledge and behavior.

The study was conducted over a 12-month period to balance scientific rigor with practical considerations such as feasibility and participant retention in rural settings. This duration aligns with prior community- and faith-based interventions in low-resource contexts that showed substantial behavioral and metabolic improvements within 1 year.^16,17,18,19,20^ In contrast, earlier programs in high-income countries (eg, the Body and Soul initiative in the US and a mosque-based intervention in Canada) reported short-term benefits over 6-week durations.^13,14^ Evidence from similar LMIC settings supports the effectiveness of year-long interventions. Our findings further demonstrate that mosque-based faith interventions can be sustained over a 12-month period, emphasizing the potential for long-term behavioral impact in rural communities.

Participants in the intervention group had a 42.5% RRR and 7.3-percentage-point ARR in T2D incidence compared with the control group, with a NNT of 14. These results are comparable to those of landmark trials such as the DPP, the Finnish DPS, and the Da Qing Study, which reported risk reductions of 42% to 58% over 2.5 to 6 years of follow-up.^6,7,8^

Importantly, the observed reduction in diabetes incidence and improvement in behavioral, clinical, and quality of life outcomes in the intervention group were achieved within 1 year, emphasizing the potential of faith-based approaches to rapidly alter health behaviors. Compared with the DMagic trial in Bangladesh, which used participatory learning and action to lower diabetes risk,^12^ our model is applied within existing religious infrastructures, suggesting advantages in cost-effectiveness and scalability.

Notably, the intervention led to clinically meaningful reductions in weight, BMI, waist circumference, FBG and 2-hour (postload) BG, and blood pressure, reinforcing the role of lifestyle changes in prediabetes management. These findings align with the Indian Diabetes Prevention Programme, which demonstrated that structured lifestyle modifications in Asian populations with prediabetes could reduce diabetes incidence by approximately 30% over 3 years.^11^ Given the cultural and socioeconomic similarities between South Asian populations, our results further support the effectiveness of culturally adapted, community-driven interventions in preventing T2D.

The beneficial outcomes observed may be partially explained by the social and spiritual influence of imams and female religious leaders, who framed health as a moral and religious obligation. Previous studies in African American churches and Canadian mosques have shown that faith-based programs can improve physical activity, dietary behaviors, and health knowledge.^13,14^ Our findings expand this evidence by demonstrating significant clinical outcomes in a Muslim-majority LMIC setting.

Although pharmacologic agents, such as metformin, pioglitazone, acarbose, and troglitazone, have demonstrated efficacy in delaying T2D onset,^26,27,28^ their implementation in rural Bangladesh may be constrained by cost, adverse effects, and access issues. In contrast, our intervention offers a sustainable, culturally congruent model that aligns with WHO recommendations for community-based diabetes prevention.

This trial also highlighted modest improvements in the control group. This finding may reflect the Hawthorne effect,^29^ wherein participants alter their behavior due to trial participation or the influence of general health advice provided at enrollment. Nevertheless, the greater effect size in the intervention group affirms the added value of structured, faith-driven engagement.

### Strengths and Limitations

To our knowledge, this is the first cluster RCT in South Asia to assess a culturally tailored, mosque-based lifestyle intervention for diabetes prevention among rural Muslim adults, addressing a critical gap in LMIC evidence. Leveraging mosque infrastructure enhanced engagement, retention, and scalability in community settings. Predefined outcomes and rigorous data collection enabled a multidimensional assessment across clinical, biochemical, behavioral, and quality-of-life domains. Sensitivity analyses further strengthened internal validity.

This study has limitations. One limitation is the potential for selection bias despite geographic separation of clusters, although district-level stratified randomization and comparable baselines reduced this risk. The small number of clusters limited subgroup power and external generalizability. The open-label design may have introduced performance bias. Self-reported behavioral outcomes were vulnerable to recall or social desirability bias. Although attrition was low and balanced, residual bias from loss to follow-up is possible. The 12-month follow-up assessed short-term effects only, and the findings may not generalize to urban or non-Muslim populations. Further research is needed to evaluate long-term sustainability and broader applicability.

## Conclusions

In this 12-month cluster RCT, a culturally adapted, mosque-based lifestyle intervention significantly reduced T2D incidence and improved metabolic and quality-of-life outcomes among adults with prediabetes in rural Bangladesh. These findings support the potential of faith-based, community-driven models as scalable, low-cost diabetes prevention strategies in low-resource settings. Further research is warranted to assess long-term effectiveness, cost-efficiency, and generalizability across broader populations.

## Notes

Supplement 1.
Trial Protocol and Statistical Analysis Plan

Supplement 2. **eTable 1.** Baseline Characteristics of Participants by Mosque Cluster **eTable 2.** Baseline Characteristics of Participants Who Completed 12-Month Follow-Up vs Those Lost to Follow-Up **eTable 3.** Primary and Secondary Outcomes at 12 Months: Intervention vs Control **eTable 4.** Sensitivity Analyses of Dropout Prediction Model **eTable 5.** Model Fit and Diagnostics

Supplement 3.
Data Sharing Statement
