# Supplementary material for: Faith-Based Lifestyle Intervention for Diabetes Prevention Among Adults in Bangladesh: A Cluster Randomized Clinical Trial
Source: JAMA Netw Open. 2025 Oct 20;8(10):e2538101. doi: 10.1001/jamanetworkopen.2025.38101 (PMC12538358; doi:10.1001/jamanetworkopen.2025.38101)
Supplement: Supplement 1. — Trial Protocol and Statistical Analysis Plan [file jamanetwopen-e2538101-s001.pdf]

Prevention of Diabetes and related NCDs through religious leaders

Version 4 (01.03.22)

Center for Global Health Research (CGHR)

Diabetic Association of Bangladesh

Room no: 233, BIRDEM Hospital

122 Kazi Nazrul Islam Avenue

Shahbag, Dhaka-1000

https://cghr-badas.org/

Project Personnel

No	Name	Role	Position
1.	National Prof A K Azad Khan	Principal Investigator	President, Diabetic Association of Bangladesh (BADAS)
2.	Prof Akhtar Hussain	Co-PI	President-Elect, International Diabetes Federation
3.	Dr. Bishwajit Bhowmik	Co-PI	Project Director, CGHR, BADAS
4.	Prof AHM Enayet Hussain	Investigator	DG, Directorate General of Medical Education (DGME)
5.	Prof Md Faruque Pathan	Investigator	Director, BIRDEM Academy, BADAS
6.	Prof Mohammed Robed Amin	Investigator	Line Director, NCDC, DGHS
7.	Dr. Faria Afsana	Investigator	Associate Professor, Department of Endocrinology, BIRDEM
8.	Dr. Tareen Ahmed	Investigator	Deputy Director, DLP
9.	Dr. Tasnima Siddiquee	Investigator	Deputy Coordinator, CGHR, BADAS
10.	Dr. Abdul Alim	Investigator	PM, Planning and Research, DGHS
11.	Dr. Fazla Alahi Khan	Investigator	PM, NCDC, DGHS
12.	Dr. Sanjida Binte Munir	Investigator	SMO, NHN, BADAS
13.	Dr. Sarwar Uddin Milon	Investigator	DPM, NCDC, DGHS
14.	Sharif Mahmood	Statistician	Assistant Professor, Department of Mathematics, University of Central Arkansas, USA

Table of Content

Sn	Topic	Page
1	Abstract	5
2	Background	6
3	Diabetes Prevention through Religious Leaders (DPRL)	7
4	Rationale of the study	7
5	Literature review	8
6	Hypothesis	9
7	Research questions	9
8	Objectives	9
9	Outcome indicators	10
10	Research methodology	10
10.1	Study design and participants	11
10.2	Study period	11
10.3	Inclusion criteria	11
10.4	Exclusion criteria	11
10.5	Study variables	11
10.6	Size of the study population and power calculation	11
10.7	Randomization and Masking	12
10.8	Flow Chart	13
10.9	Intervention	13
11	Data collection	15
12	Data analysis	19
13	Health economic analysis	20
14	Ethical approval	20
15	Funding of the study	21
16	Staff of the study	21
17	Responsibilities of the staff	21
18	Governance and quality contol	22
19	Reporting adverse events	23
20	Indemnity	23
21	Future perspective	23
22	Conflict of interest	23
23	Report writing of the project	23
24	Dissemination of the results	23

25	Time frame	24
26	Budget	24
27	References	26

1. Abstract:

Background: Type 2 diabetes mellitus (T2DM) and related non-communicable diseases (NCDs) are significant public health challenges in Bangladesh, with a high prevalence among adults. Culturally tailored, faith-based interventions led by religious leaders have shown promise in promoting healthy behaviors and preventing T2DM. This study will evaluate the effectiveness of a faith-based lifestyle intervention delivered by mosque imams in reducing T2DM incidence and improving cardiometabolic outcomes among high-risk individuals in rural Bangladesh.

Objectives: The primary objective will be to assess the impact of a faith-based lifestyle intervention on the incidence of T2DM among high-risk individuals with prediabetes. Secondary objectives will include evaluating changes in cardiometabolic parameters, quality of life, and diabetes-related knowledge.

Design: A cluster-randomized controlled trial (RCT) will be conducted across eight mosques in rural Bangladesh. Mosques will be randomly assigned to either the intervention or control group. The intervention will consist of religious sermons promoting healthy behaviors, dietary guidance, and physical activity, while the control group will receive standard health education.

Study areas: The study will be conducted in eight mosques located in the districts of Manikganj, Narshingdi, Munshigonj, Dhaka, and Tangail. Four mosques will serve as intervention sites (Shibaloy, Monohardi, Raipura, and Dhanbari), and four will serve as control sites (Singair, Keraniganj, Sreenagar, and Sakhipur).

Study duration: 12 months (April 2022 to April 2023).

Target population: A total of 824 participants aged 25–65 years with prediabetes (based on WHO criteria) will be enrolled. Participants will be permanent residents of the study areas and willing to participate for the entire study duration.

Statistical analysis: The primary outcome will be the cumulative incidence of T2DM at 12 months. Secondary outcomes will include changes in weight, body mass index (BMI), blood pressure, blood glucose, lipid profiles, and quality of life. Data will be analyzed using intention-to-treat analysis, with comparisons between groups made using chi-square tests, t-tests, and mixed-effects models.

Conclusion: This study will provide evidence on the effectiveness of a faith-based lifestyle intervention in preventing T2DM and improving cardiometabolic health in a low-resource setting. The findings will inform scalable and culturally relevant strategies for diabetes prevention in Bangladesh and similar contexts.

Keywords:

Diabetes prevention, faith-based intervention, religious leaders, lifestyle modification, Bangladesh.

Study Coordination: Diabetic Association of Bangladesh.

2. Background

Diabetes (T2DM) is now considered a significant public health burden in Bangladesh. International Diabetes Federation (IDF) reported that 13.1 million people living in Bangladesh had T2DM in 2021, and of them, 43% were unaware of their diabetes (T2DM) status [1]. Bangladesh at present is in the 8th position in the world by the total number of people with T2DM. Older age, affluent socioeconomic status, carbohydrate-rich foods, physical inactivity, obesity, smoking, HTN, dyslipidemia, increased age at pregnancy, and depression are significant risk factors for T2DM [2-4]. Studies have also shown that people with T2DM in Bangladesh receive two times more inpatient treatment days and require one and half times more outpatient visits and more than nine times more medications than normal individuals [5]. Overall, a person with T2DM in Bangladesh spends nine percent of their annual household income managing the disease. The total annual per capita expenditure on medical care is six times higher for people with T2DMs (US$635 vs. US$104, respectively). Using productivity-adjusted life years (PALY), it is projected that more than nine million PALYs (20.4%) are attributable to having T2DM. In Bangladesh, the loss in PALYs linked to a total of US$97.4 billion lost (US$16 987 per person) in gross domestic product [6, 7].

T2DM is a complex metabolic disorder. Before diagnosis, it normally passes through a prolonged dormant period and remains untreated. Studies have shown that approximately 30–50% of people with T2DM usually presented with one or more micro- or macrovascular complications at the time of diagnosis [8-10]. For these reasons, early identification of people with undiagnosed T2DM or those at an increased risk for developing T2DM has been recommended to improve outcomes. Several randomized clinical trials have also shown that lifestyle intervention strategies can prevent and delay the progression of T2DM among high-risk individuals, which is highly cost-effective [11-15].

The majority of people (89%) in Bangladesh are Muslim. Mosques are the common meeting place for adult Muslims in Bangladesh, and religious leaders (Mosque Imams) are respected and influential figures in the Muslim community. People seek advice from the Mosque Imams about all sorts of matters and trust them with personal and family matters. Not only that, in the Islamic tradition, a religious sermon (Khutbah) serves to deliver words of exhortation, instruction, or command at gatherings for worship in the mosque at weekly (congregation prayer on Friday) and annual (Eid) rituals. Powerful messages on a healthy lifestyle already exist in Islam. Therefore, there is a wonderful opportunity to seek the influence of Imams in creating community awareness about the prevention of T2DM and other NCDs and helping educate people to take proactive action and change their lifestyles. To date, we have not estimated the value that could be gained from investing in faith-based lifestyle interventions targeting the adult Bangladeshi population.

To address this gap in the evidence and inform decision-making for T2DM prevention among the Bangladeshi population, this study will evaluate the effect of faith-based (religious leaders) lifestyle intervention to prevent T2DM in high-risk individuals using a prospective randomized control study design.

3. Diabetes Prevention through Religious Leaders (DPRL) [16]

Considering the influence of religious leaders, BADAS has started a prevention program, "Prevention of Diabetes through Religious Leaders," for the last three years to improve community awareness of a healthy lifestyle for prevention and care of diabetes and other NCDs through religious leaders. Initially, it was funded by World Diabetes Foundation. It is now a joint community awareness program of BADAS, the Islamic Foundation of Bangladesh (Ministry of Religious Affairs), and the Non-Communicable Disease Control (NCDC) program of DG Health (Ministry of Health and Family Welfare).

100 Masque Imams from 100 Upazilas of Dhaka and Mymensingh division received training, and BADAS has already established 100 diabetes corners in their Mosques. Besides Friday Sermon, Mosque Imams usually check blood glucose, blood pressure, anthropometric measurement and provide basic education on a healthy lifestyle to prevent T2DM and related diseases in their diabetes corners. They also charge 40 BDT for using the health package. As part of the program, Each Imam has a female assistant (she may be his wife, sister, daughter, or any woman appointed by the local Mosque committee) for improving female participation in their community. About 30000 people have already taken care of these centers. Islamic Foundation, Ministry of Religious Affairs; Non-communicable Disease Control program of DG Health, Ministry of Health and Family Affairs; National Mosque of Bangladesh, Bangladesh Madrasah Education Board, and Befaqul Madarishil Arabia Bangladesh have approved this program, and the religious sermon (Khutbah) for its nationwide application. Islamic Foundation has also given written instruction to three lacs registered mosques throughout the country for using this Khutbah for creating community awareness on a healthy lifestyle. Besides these, a total of 100000 Mosque Imams received the printed Khutbah for building community awareness. It is now the biggest platform for creating community awareness on a healthy lifestyle to prevent diabetes and related NCDs.

4. Rationale of the study

Like other South Asian (SA) countries, Bangladesh is also facing an increased health challenge associated with rapid economic transition, migration from traditional rural to more urban locations, and changes in their dietary and physical activity practices [17-19]. An increased predisposition to non-communicable diseases (NCDs) is an adverse consequence of these changes, with increased prevalence of obesity and central adiposity leading to an increased risk of T2DM. The Government of Bangladesh has adopted the WHO Global Action Plan for the Prevention and Control of NCDs 2013–2020, and they have further committed to universal health coverage (UHC) by 2030. Although the country has a national action plan for facing the epidemiological transitions of T2DM, countrywide implications have received less attention.

Early identification of people with undiagnosed T2DM or those at an increased risk for developing T2DM has been recommended to improve outcomes. Several randomized clinical trials have also shown that lifestyle intervention strategies can prevent and delay the progression of T2DM among high-risk individuals, which is highly cost-effective. One Bangladeshi study showed that overall, US$297 could be saved annually by

preventing only one case of T2DM [20]. The Government should prioritize T2DM prevention and control programs, such as creating mass awareness and changing lifestyle habits through well-designed public health programs.

The majority of people (89%) in Bangladesh are Muslim. Mosques are the common meeting place for adult Muslims in Bangladesh, and religious leaders (Mosque Imams) are respected and influential figures in the Muslim community. People seek advice from the Mosque Imams about matters and trust them with personal and family issues. Not only that, in the Islamic tradition, a religious sermon (Khutbah) serves to deliver words of exhortation, instruction, or command at gatherings for worship in the mosque at weekly (congregation prayer on Friday) and annual (Eid) rituals. Along with religious issues, religious leaders also provide solutions for the different problems arising in society through Khutbah. Powerful messages on a healthy lifestyle already exist in Islam. Therefore, there is a wonderful opportunity to seek the influence of Mosque Imams in creating community awareness about preventing T2DM and other NCDs and helping educate people to take proactive action and change their lifestyles.

In Bangladesh, the Diabetic Association of Bangladesh (BADAS) has successfully included religious leaders to influence the improvement of community awareness about the prevention of T2DM - "Diabetes Prevention through Religious Leaders" and the prevention of GDM and future T2DM "Preconception Care through Religious Leaders" [16, 21]. The Bangladesh DMagic trial reported that community mobilization using the participatory learning and action (PLA) approach improved knowledge and awareness about T2DM and significantly lowered the prevalence of T2DM and intermediate hyperglycemia [22]. Therefore, it is vital to estimate the effectiveness of faith-based lifestyle interventions targeting the adult Bangladeshi population for policy implications and design suitable preventive interventions. Data on the current state of T2DM and its prevention in rural areas is also important because about 62.6% of the total population lives in rural communities of Bangladesh.

5. Literature review: (will describe the previous and ongoing works related to this study)

We searched PubMed, CINAHL, EMBASE, Web of Science, and Google Scholar for systematic reviews and published original studies published up to May 2021, with a particular focus on lifestyle intervention for the prevention of T2DM through Religious Leaders in low and middle-income countries. We used the search terms "T2DM", "diabetes prevention," "lifestyle interventions," "religious leaders," "peer education," and "community participation." There were no language restrictions used. Because we wanted to understand both the nature of existing interventions and their effectiveness, we were interested in a range of studies, including systemic reviews, randomized controlled trials, pilot studies, and case-control studies.

Good evidence exists for group support, and peers support lifestyle interventions to prevent or delay the onset of T2DM [22]. Evidence supports community and peers' involvement as a cost-effective means of promoting lifestyle changes in high-income settings, but research in resource-poor settings is lacking. Evidence on the

effects of community mobilization on T2DM and related risk factors among the general population, as opposed to high-risk individuals, is yet to emerge from low-income or middle-income countries.

Several recent efforts to reach high-risk populations, such as African Americans, have focused on delivering lifestyle interventions through faith-based institutions (FBO) [23, 24]. Findings indicate FBOs may be a promising avenue for providing diabetes self-management education (DSME) to Black Americans. Informed by the results, a focused discussion on advancing the science of faith-based interventions to expand the delivery of DSME to Black Americans with T2DM is provided [25-27].

Studies have shown that Islamic religious leaders (IRLs) can influence health education, health promotion, and positive health outcomes among their communities. They have played a significant role in creating public awareness of reproductive health, HIV/AIDS, immunization, and family planning and played an important role in paving the way for promoting a healthy lifestyle [28-29]. Religious decrees (fatwa) issued by Muslim scholars guide diabetic patients in various fields from Insulin injection, other medications, fasting in Ramadan, and even novel treatments such as therapeutic use of stem cells.

Study findings reveal that 1) culturally sensitive, behaviorally oriented interventions incorporating social support are needed to achieve positive health outcomes. Findings indicate FBOs may be a promising avenue for delivering DSME, 2) IRLs are effective social agents for change, and that the educational interventions can be a useful and effective strategy to encourage IRLs to cooperate with health providers and promote public health among their communities and 3) religion should be explored as a potential tool to reach out on facts while doing away with erroneous beliefs about T2DM.

6. Hypothesis:

- A faith-based lifestyle intervention delivered by mosque imams will significantly reduce the incidence of type 2 diabetes mellitus (T2DM) among high-risk individuals with prediabetes in rural Bangladesh over a 12-month period, compared to a control group receiving standard health education.

7. Research questions:

- Does a faith-based lifestyle intervention delivered by mosque imams reduce the incidence of type 2 diabetes mellitus (T2DM) among high-risk individuals with prediabetes in rural Bangladesh over a 12-month period, compared to a control group receiving standard health education?

8. Objective:

8.1 Primary objectives:

- To evaluate the effectiveness of a faith-based lifestyle intervention in reducing the incidence of T2DM among high-risk individuals with prediabetes over a 12-month period.

8.2. Secondary objective:

- To assess changes in cardiometabolic parameters, including weight, BMI, blood pressure, blood glucose, and lipid profiles.

- To assess changes in dietary habits and physical activity levels.
- To evaluate improvements in quality of life and diabetes-related knowledge among participants.
- To determine the cost-effectiveness of the intervention.

9. Outcome Indicators

9.1 Primary outcomes:

- Cumulative incidence of T2DM at 12 months.

9.2 Secondary outcomes

- Changes in cardiometabolic risk factors (weight, BMI, BP, lipid profile, glycaemic control).
- Improvements in diabetes knowledge and self-care behaviors.
- Changes in physical activity and dietary habits.
- Cost-effectiveness assessment.

10. Research methodology

10.1 Study design and participants

This cluster-randomized controlled trial (RCT) will be conducted across eight mosques in rural villages of five districts in Bangladesh: Manikganj, Narshingdi, Munshigonj, Dhaka, and Tangail. The study will target rural areas to address the unique challenges faced by these communities. Participants will be randomly allocated to either the intervention or control group at the cluster level (mosques). Eight mosques will be selected using a simple random procedure, with four assigned to the intervention group and four to the control group. Mosques will be randomly assigned as intervention or control sites using block randomization to ensure geographic diversity and balance across groups. Randomization lists will be generated centrally and concealed from field teams to minimize bias. Intervention centers will be in Shibaloy, Monohardi, Raipura, and Dhanbari, while control centers will be in Singair, Keraniganj, Sreenagar, and Sakhipur. All mosques are recognized by the Islamic Foundation of the Ministry of Religious Affairs and have an education center for learning the Holy Quran.

Participants will be recruited using the most recent voter list and age distribution data for Bangladesh. Each site will have an estimated population of 2,500 to 3,000 individuals, from which 1,500 individuals aged 25 to 65 years will be invited to participate. According to Bangladesh's age distribution, 45–47% of the population falls within this age group [30, 31]. The target recruitment goal is to enroll up to 108 participants from each mosque.

Recruitment will initiate at each mosque 4 weeks before the introduction of the intervention. The Imams will discuss and convey the message of a healthy lifestyle including diet and exercise during the Friday sermon and use a standardized script to encourage congregational participation in practice. They will distribute investigator-developed flyers to congregational participants to promote the study objectives. They will also discuss the importance of female participation as part of the intervention. Members of the research team will also attend Friday sermons to assist with recruitment and provide information regarding the study.

Any men and non-pregnant women aged 25 years and older with an individual's diabetes risk score ≥9 and diagnosed prediabetes (IFG/IGT) as per WHO recommended diagnostic value will be the units of analysis. Project members will use the DPRL diabetes corner situated near the mosque for the screening, intervention, and further follow-up of male participants. For females, project members will use a house nearby the selected mosques. We will use an Imam's woman assistant in each center for the requirements of women participants.

10.2 Study period: 12 months (April 2022 to April 2023).

10.3 Inclusion Criteria

- All individuals aged 25-65 years with diabetes risk score ≥9 [32] and diagnosed with prediabetes (IFG/IGT) as per WHO recommended diagnostic value [33]. These are the primary inclusion criteria.
- Both Gender
- Permanent residents
- Willing to participate and available for 12 months of study

10.4 Exclusion criteria

- Pregnant women or planned pregnancy and lactating mothers
- Known case of T2DM
- Individual diagnosed with acute physical or mental illness at the time of screening

10.5 Study variables

- **Socio-demographic variables:** age, sex, economic status, education status, smoking habit, lifestyle (physical activity and food habit).
- **Anthropometric variables:** height, weight, body mass index (BMI), waist circumferences (WC), waist-height ratio (WHR), and waist-height ratio (WHtR).
- **Clinical & Biochemical variables:** blood pressure, fasting blood glucose (FBG), 2-hour blood glucose, HbA1c, fasting lipid profile, ECG and fundus photography.
- **Quality of Life**: Assessed using the EuroQol-5D-5L scale and EuroQol-VAS score.
- **Diabetes Knowledge**: Assessed through structured questionnaires.
- **Economic Variables:** employment status, productivity loss (income and days/hours of work lost), out-of-pocket payments (OPP), the opportunity cost of time (average wage and time), borrowing/selling assets, expenditure.

10.6. Size of the study population and power calculation

The sample size for this cluster-randomized controlled trial (RCT) was calculated to detect a 50% reduction in the incidence of type 2 diabetes mellitus (T2DM) in the intervention group compared to the control group. This assumption was based on evidence from previous landmark lifestyle intervention trials, such as the Diabetes Prevention Program (DPP) in the United States and the Finnish Diabetes Prevention Study (DPS), which reported a 58% reduction in T2DM risk among high-risk individuals.

Key Parameters for Sample Size Calculation:

1. Primary Outcome: A 50% reduction in T2DM incidence in the intervention group compared to the control group.

2. Power: 80% power to detect the effect.
3. Significance Level: 5% ($\alpha = 0.05$).
4. Intraclass Correlation Coefficient (ICC): 0.05 to account for clustering within mosques.
5. Dropout Rate: 50% to account for potential loss to follow-up.

The sample size calculation used the following formula for cluster-randomized trials:

$$n=2\cdot(Z_{\alpha/2}+Z_{\beta})2\cdot p\cdot(1-p)\cdot DE/\ (p1-p2)^2$$

Where:

- $Z_{\alpha/2=}$1.96 (for a 5% significance level).
- Z_{β}=0.84 (for 80% power).
- p= overall event rate (T2DM incidence in the control group).
- p1= event rate in the control group.
- p2= event rate in the intervention group (50% reduction).
- DE= design effect, calculated as $1+(n-1)\cdot\rho 1+(n-1)\cdot\rho$, where ρ=0.05(ICC).

Assumptions:

- The 5-year cumulative incidence of T2DM in Bangladesh was 16.4 per 1,000 person-years [34].
- A 50% reduction in T2DM incidence in the intervention group was assumed, based on previous trials.
- The design effect (DE) was calculated as 6.1, accounting for clustering within mosques.

Calculation:

1. Number of Participants per Arm:
   - Based on the formula and assumptions, 412 participants per arm were required to detect a 50% reduction in T2DM incidence with 80% power and a 5% significance level.
2. Total Sample Size:
   - To account for a 50% loss to follow-up, the total sample size was increased to 824 participants (412 in the intervention group and 412 in the control group).
3. Number of Clusters:
   - Participants were recruited from eight mosques, with approximately 103 participants per mosque.

10.7. Randomization and Masking

Participants will be randomized after the project research team determined that they:

1. Will demonstrate a satisfactory interest in joining lifestyle intervention during the run-in.
2. Will express willingness to continue in the trial.
3. Will meet all inclusion/exclusion criteria.
4. Will provide all baseline data.

After exclusions, 824 participants will be allocated at random in a 1:1 ratio to an intensive lifestyle intervention to promote increased physical activity and dietary modification or to minimal lifestyle advice to the control group. Randomization lists will be generated and supplied by the coordinating center, and staff who will perform the baseline measurements will have no access to the randomization lists. Neither staff delivering the intervention nor participants will be masked to the study arm.

10.8 Flow Chart

Slection of eight mosques located in the districts of Manikganj, Narshingdi, Munshigonj, Dhaka, and Tangail

Individuals aged 25-65 years with a diabetes risk score (BDRS) of ≥9 points will be invited to participate in the program

Advise OGTT

Normal
(Exclude from the study)

Prediabetes (IFG/IGT)
(Include in the study) (n=824)

Diabetes
(Exclude from the study)

Intervention Group (n=412)

Control Group (n=412)

During Registration

During Registration (intervention group)

Blood tests + Baseline data collection + **recommended intervention** + Follow-up monthly / 1st 4 months

During Registration (control groups)

Blood tests + Baseline data collection

Follow-up

At 4 months

Blood tests + data collection + **recommended intervention** + Follow-up monthly/ 2nd 8 months

At 4 months

Blood tests + data collection

Follow-up

At 12 months

Blood tests + End line data collection

At 12 months

Blood tests + End line data collection

10.9 Intervention

The faith-based intervention will combine religious teachings with evidence-based health strategies, comprising the following key components:

1. **Religious Sermons (Khutbah):**
    - Imams will deliver sermons during Friday prayers to male participants, while female assistantswill conduct group sessions to deliver similar messages to female participants.
    - These sessions will emphasize healthy lifestyle practices and incorporate culturally relevant religious principles to inspire behavioral change.

- To support their engagement, Imams and their female assistants will be incentivized with payments for their participation in delivering the sermons.

2. **Diet and Lifestyle Guidance:**
   - Participants will receive practical advice to reduce rice consumption, limit the use of extra salt, oil, and sugary foods, and incorporate more vegetables and fruits into their diet.
   - Visual aids, such as the plate model, will be utilized to illustrate balanced eating habits effectively.
3. **Physical Activity:**
   - Male participants will walk with Imams after morning prayers, while female participants will engage in walking sessions with female assistants after afternoon prayers.
   - The goal will be to achieve at least 30 minutes of daily physical activity.
4. **Performance Monitoring:**
   - Trained volunteers will document participants' dietary patterns, physical activity levels, blood pressure, and anthropometric measurements (including weight, waist, and hip circumference) in individual guidebooks.
   - These records will be reviewed during sessions to discuss progress and set future goals.

Each intervention session will last approximately two hours and will be conducted monthly, supplemented by detailed assessments at 4 and 12 months.

Intervention tool development

The following interpersonal education and communication (IEC) materials developed by the DPRL program of BADAS will be used for this purpose-

- Khutbah
- Guidebook including dietary and exercise diary
- Flip chart
- Poster & leaflets

Intervention session

Twelve (12) sessions for each subject will be carried out for the next 12 months based on the following schedule-

Serial	Visits	Duration	Tasks
1	*Initial screening*	*2 -3 hours*	*Lab tests only*
**2**	**Inclusion 0 weeks**	**2 – 3 hours**	**Motivation and Counseling**
3	1 month after inclusion	1 hour	Motivation and counseling
4	At 2 months	1 hour	Motivation and counseling
5	At 3 months	1 hour	Motivation and counseling
**6**	**At 4 months**	**3 hours**	**Motivation and Lab Tests**
**7**	At 5 months	1 hour	Motivation and counseling
**8**	At 6 months	1 hour	Motivation and counseling
**9**	At 7 months	1 hour	Motivation and counseling
**10**	At 8 months	1 hour	Motivation and counseling
11	At 9 months	1 hour	Motivation and counseling
12	At 10 months	1 hour	Motivation and counseling

13	At 11 months	1 hour	Motivation and counseling
**14**	**At 12 months**	**3 hours**	**Motivation and Lab Tests**

Control group

The subjects in the control group will be educated regarding general principles of a healthy lifestyle based on the religious sermon (khutbah) that benefits T2DM prevention and informed about the current evidence showing that the lifestyle intervention is effective for T2DM prevention during the ''run-in'' period. After the inclusion and collection of baseline data, they will visit the study site at 4 months and 12 months for the collection of anthropometric, clinical, and laboratory investigations.

11. Data collection

Phase 1: Pre-screening contact

The Imams will discuss and convey the message about the study program to the congregational participants during the Friday sermon. Any interested individual (including both male and female) aged 25 -65 years will be invited for risk assessment. A group of trained personnel of BADAS will assess the participant's risk for T2DM by using the non-invasive risk score developed by BADAS. All individuals aged 25-65 years with a diabetes risk score ≥9 will participate in the screening visit. Individuals will be advised to fast overnight for at least 8-14h before the screening visit.

Phase 2: Sample survey and collection of other data

Prior to initiating the survey, the study team will organize **a** comprehensive training program for Imams and female assistants at BADAS's tertiary hospital (BIRDEM Hospital) in Dhaka, complemented by hands-on training sessions at the study sites. Local volunteers will be recruited and will receive standardized training covering data collection, intervention implementation, and participant monitoring. The training curriculum will include modules on diabetes prevention, lifestyle modification, and the effective use of educational materials. To ensure adherence to study protocols, volunteers will be closely supervised by the specialized research team.

A multidisciplinary team will be formed, consisting of four diabetologists, four dieticians, two health educators, one IT specialist, two eye technicians, and six phlebotomists. This team will be responsible for overseeing data collection at baseline, 4 months, and 12 months. The team will undergo an intensive weeklong training to standardize data collection procedures. Compliance with study protocols will be rigorously monitored through regular audits conducted by data safety officers, while independent evaluators, blinded to group assignments, will carry out outcome assessments to reduce bias and enhance the reliability of findings.

Study plan/timetable

The proposed study will be implemented following the timetable as below:

Procedure	Visit 1	Visit 2	Visit 3
Timing	At screening	4 months	12 months
Informed consent	X		
Check eligibility criteria	X		
Record basic demographic data	X		
Dietary questionnaire	X	X	X

Health questionnaire	X	X	X
Knowledge questionnaire	X	X	X
Economic questionnaire	X		
Blood pressure	X	X	X
Anthropometric measurements	X	X	X
Foot examination	X	X	X
Collect venous blood samples	X	X	X
ECG	X	X	X
Randomization	X		
Run-in	X		
Adverse event recording	X	X	X

Informed consent:

Full informed consent will be obtained. All potential participants will be given adequate time to read participant information sheets and had a chance to ask questions to satisfy any queries they have before consent. Staff members taking consent must assess whether potential participants have fully understood the information they provided before taking consent. Those who are not thought to be fully informed will not be included in the trial. All staff members taking consent will be trained in this procedure.

Registration

This will be included informed consent and completion of a detailed medical eligibility questionnaire. The eligibility screening questionnaire will be based on the inclusion/ exclusion criteria described above. If eligible, subjects have to finish the baseline survey at the same time. At the baseline survey, all participants will complete an interview-administered questionnaire and undergo a physical examination. They will also complete the 24-h food records using dietary record collections will be taught by trained investigators.

Run-in

A one-week ‘‘run-in’’ period followed the baseline survey during which two classes on general principles of lifestyle intervention for the prevention of T2DM will be taught. The specific lifestyle intervention program will not begin during this period. If an individual diagnosed with T2DM (fasting glucose ≥7.0 mmol/l or 2-h glucose ≥11.1 mmol/l) in our baseline OGTT test, we would exclude them from the present study at this period.

Development of interviewer-administered questionnaires

Before the study, a panel of experts will be agreed on a structured questionnaire after thorough discussions and also in the light of surveys conducted in previous studies. The panel will be included an endocrinologist, a diabetologist, a statistician, and a public health expert. The questionnaire will be developed by consulting existing information and clinical practices. The questionnaire will be developed in the local language of Bangla. Before the start of the study, pretesting of the questionnaire will be conducted to test its feasibility and reliability. As a result, a few minor modifications will be made after the pilot testing. Trained investigators will be collected data after face-to-face interviews with the participants. To assuring quality control, intensive training will be provided for the investigators to reduce bias as much as possible.

Sample survey

Upon arrival at the field sites, different sets of investigations and physical examinations will be done for each of the subjects taking part in the study. At first, an initial fasting blood sample will be taken. Then all the subjects will be given 75-gram oral glucose to drink and will be requested to wait for 2-hours for the second

blood sample collection. During this 2-hour waiting time, socioeconomic and demographic information, and parental and personal health histories will be verbally obtained using standardized questionnaires. After completion of the interview, the anthropometric measurements including height, weight, hip and waist circumference (WC) will be taken. Also, blood pressure (BP) will be recorded at this time followed by a physical examination. These will be carried out through trained personnel. After 2 hours, a second blood sample for OGTT will be collected.

Anthropometrical measurements

Anthropometric measurements including height, weight, and waist and hip circumferences will be taken with the subjects wearing light clothes and without shoes. Weight will be recorded to the nearest 0.1 kg using electronic digital LCD weighing machines (Best Deluxe Model; Bathroom, Dhaka, Bangladesh) placed on a flat surface. The scales will be placed on a flat surface and calibrated using a standard (20 kg) each day. Height will be taken while the subjects stood in the erect posture, touching the occiput, back, hip, and heels on a straight measuring wall, while the subjects looked straight ahead. Body mass index (BMI) will be calculated as the weight (Kg) divided by the square of the height (m^2). WC will be measured by placing a plastic tape horizontally midway between the lower border of the ribs and the upper border of the iliac crest on the mid-axillary line. Hip circumference will be measured to the nearest centimeter at the greatest protrusion of the buttocks. Waist-hip ratio (WHR) will then be calculated from WC (cm) and height (cm), respectively.

Measurement of blood pressure

Special precautions will be taken to reduce the variation of BP value with resting BP. BP will be measured by 1) ensuring the participant rested for 10 minutes before the BP being measured, 2) using standard cuffs for adults fitted with a standard mercury sphygmomanometer, and 3) placing the stethoscope bell lightly over the pulsatile brachial artery on the right arm. Blood pressure will be recorded to the nearest 2 mmHg from the top of the mercury meniscus. Systolic pressure will be recorded at the first appearance of sounds, and diastolic pressure will be measured at phase V, that is, the disappearance of sounds. BP will be measured in both the sitting and standing position. Two readings will be taken 5 minutes apart, and the mean of the two will be taken as the final BP reading of the individual.

Foot examination

The dorsalis pedis and posterior tibialis arteries will be palpated to identify peripheral vascular disease. Additionally, a 10-gram nylon monofilament and a 128 Hz tuning fork will be used to identify peripheral neuropathy.

Biochemical examination

At first, an initial blood sample will be taken from the fingertip (70 μL) by using a point of care machine to estimate the fasting plasma glucose (FPG), HbA1c, and fasting lipid profile. Then all the subjects will be given 75-gram oral glucose to drink and requested to wait for 2-hours for a second blood sample collection. During this 2-hour waiting time, they will be interviewed for the collection of socio-demographic information. Pre-formulated questionnaires will be used for this purpose. Each participant will be interviewed for approximately 25 to 30 minutes to complete the questionnaire. After completion of the interview, the anthropometric measurements and foot examination with monofilament, and tuning fork will be recorded. In addition, blood pressure will be recorded at this time. After 2 hours, a second blood sample for OGTT will be done by glucometer.

The time frame of biochemical assessment

Lab test	Timing		
	0 month	4 months	12 months
Fasting blood glucose	x	x	x
2h blood glucose	X	X	X
HbA_{1C}	X	X	X
Total cholesterol	X	X	X
Triglycerides	X	X	X
HDL-Cholesterol	X	X	X
LDL-Cholesterol	X	X	X
ECG	X	X	X
Fundoscopy	X	X	X

Methods and specification of the machines for the various biological tests

**Test**		**Sample**		**Method**		**Specification of Machine**
						**Name of the analyzer**
OGTT<br>• FBG<br>• 2h AG		Capillary whole blood		Glucose dehydrogenase		Alere G1 (South Korea)
T-Chol		Capillary whole blood		Enzymatic method		Alere Cholestech LDX@ (USA)
Tg		Do		Enzymatic method		Do
HDL		Do		Enzymatic method		Do
LDL		DO		Enzymatic method		Do
HbA_{1C}		Capillary whole blood		Borronate Affinity		AFINION 2 (Norway)

Fundus Photography

We will collect retinal photographs using a non-mydriatic digital fundus retinal camera and using a two-field imaging protocol (Canon CR6 non-mydriatic retinal fundus camera). A retinal fundoscopy machine will be used for taking retinal images. An Artificial Intelligence (AI) based algorithm will also be developed using retinal images to identify Diabetic Retinopathy (DR) cases.

Definition of terms

- DM will be defined as if FPG ≥7.0 mmol/L or 2hPG ≥11.1 mmol/L. Prediabetes will be defined as FPG ≥6.1 mmol/l to <7.0 mmol/l (impaired fasting glycemia, IFG) and 2hPG ≥7.8 mmol/l to <11.1 mmol/l (impaired glucose tolerance, IGT). Normal glucose tolerance (NGT) will be defined as FPG <6.1 mmol/l and 2hPG <7.8 mmol/l. For screening of NGT, prediabetes, and DM, diagnostic criteria of WHO in 2006 will be used [33].

- Cut off points for general obesity for both sexes will be defined as BMI of ≥25 kg/m^2 [35] and central obesity including WC for men and women will be ≥90 and ≥80 cm [34], and WHR for men ≥0.90 and women for ≥0.80 [36], respectively.
- Hypertension will be defined as systolic blood pressure (SBP) of 140 mm Hg or diastolic blood pressure (DBP) of 90 mm Hg or current treatment with antihypertensive medication [37].
- Cut-off values for serum lipid profiles will be high cholesterol (T-Chol) ≥5.0 mmol/l, high triglycerides (Tg) ≥1.7 mmol/l, high LDL-C ≥3.4 mmol/l and low HDL-C <1.04 mmol/l (for men) and <1.3 mmol/l (for women) [38].
- Dyslipidaemia will be defined as serum triglycerides ≥1.70 mmol/L for both sexes and HDL-C <1.04 mmol/L for men and <1.3 mmol/L for women [37].
- Bangladesh Diabetes Risk Score (BDRS) will be developed based on multiple logistic regression analysis using five simple parameters namely age (≤30 = 0, 31-40 = 3, ≥41 = 4), sex (female = 0, male = 2), BMI (<25 kg/m^2 = 0, ≥25 kg/m^2 = 2), WHR (m <0.90; f <0.80 = 0, m ≥0.90; f ≥0.80 = 5), and presence of HTN (no = 0, yes = 2). Subjects with a BDRS of <5 was categorized as low risk, 5 to 9 as medium risk and >9 as high risk for T2DM [32].
- Smoking habits will be classified as either current or non/ex-smoker.
- Socio-economic condition will be classified as low (<5000 Bangladeshi Taka [BDT, 1 USD = 84 BDT]), medium (5000-1000 BDT) and high (>10000 BDT) based on the monthly expenditure.
- Physical activity will be graded on a 1-3 ordinal scale based on leisure-time walking duration: light (<30 minutes), moderate (30-60 minutes), and heavy (>60 minutes). For data analysis, these categories will be transformed into a binary variable: inactive (grade 1: <30 minutes) and active (grades 2 and 3: ≥30 minutes) [39, 40].
- Diabetes-related knowledge will serve as a validated instrument assessing diabetes-related knowledge. There will be 11 questions, where correct answers will earn 1 point, while incorrect or “don’t know” responses will earn no points. This study will use a percentage score to evaluate knowledge [41].
- Health-Related Quality of Life of the participants will be assessed using the validated EuroQol-5D-5L scale and EuroQol-VAS score [42].

12. Data analysis

A data safety officer will monitor the study. The primary and secondary outcomes will be analysed using the intention-to-treat approach. Differences in baseline characteristics and clinical outcomes between the two groups will be evaluated using two-sample t-tests for continuous variables and chi-square tests for categorical variables.

For the primary outcome, the cumulative incidence of T2DM will be compared between the two groups using a log-rank test with random effects for intercept to account for pre-post correlations within participants. The intervention effect will be assessed at each time point. For secondary outcomes, which will be continuous variables, repeated-measure linear mixed models will be used to assess differences between groups over time.

The incidence rate of diabetes will be calculated by dividing the number of new diabetes cases by the total person-time of follow-up, expressed per 1,000 person-years. This method will account for participants with differing lengths of follow-up, including those who drop out or are lost to follow-up. Incidence rates will be

reported as events per 1,000 person-years, along with 95% confidence intervals (CIs), to provide a measure of precision and enable comparisons between groups.

The cumulative incidence of T2DM over the 12-month follow-up period will be estimated using Kaplan-Meier survival analysis. Differences between the survival curves will be tested using the log-rank test to determine statistical significance. To quantify the intervention effect, hazard ratios (HRs) with 95% CIs will be calculated using a Cox proportional hazards regression model, which will adjust for the time to diabetes development. Participants who do not develop T2DM or are lost to follow-up will be censored at the time of their last follow-up, ensuring accurate risk estimation.

For intervention measures, chi-square tests will be used to compare event rates between the intervention and control groups. Absolute risk reduction (ARR) and relative risk reduction (RRR) will be calculated, with risk ratios used to quantify the relative difference in event rates between the two groups. Confidence intervals for ARR and RRR will be computed to estimate the precision of the findings. The number needed to treat (NNT) to prevent one case of T2DM will be determined to evaluate the practical impact of the intervention.

Changes in biophysical, clinical, and laboratory parameters will be analyzed using a difference-in-differences approach, comparing changes within and between the intervention and control groups over time. This method will account for baseline differences and provide a robust estimate of the intervention effect. The difference-in-differences approach will be chosen because it isolates the intervention effect by controlling for time-invariant differences between groups and common trends over time. All analyses will be adjusted for clustering at the mosque level using mixed-effects models to account for intraclass correlation. Statistical significance will be set at $p < 0.05$, and all analyses will be performed using STATA version 17.0 (StataCorp, College Station, TX, USA) and the statistical package SPSS (PASW Statistics 20).

13. Health economic analysis

Economic analyses (cost-efficacy/effectiveness and budget impact analyses) of those interventions implemented will be carried out under a limited societal perspective. Thus, direct (medical and non-medical), as well as indirect costs, will be considered. Direct medical costs will be collected alongside other clinical data generated in the trial. Direct non-medical and indirect costs will be collected by questionnaire.

We will focus on the impact of the intervention on economic outcomes including employment status, productivity loss (lost productivity due to ill health), expenditure, and assets (based on an asset index which will be computed from questions about ownership of assets, housing type and access to services). We will use two-stage models. First, we will evaluate the distributional impact of the intervention using difference-in-differences (DID) analysis to compare outcomes for the intervention group with the control group. In the second part, to evaluate the impact on economic outcomes, we will regress the predicted measure of T2DM morbidity from the first stage, on the economic outcome variables (e.g. productivity loss, catastrophic health spending). For each outcome, we will run appropriate regressions. We will take account of socio-demographic and other control variables including gender and will run sensitivity checks to ensure the robustness of our models.

14. Ethical approval:

The study will be conducted according to the guidelines laid down in the Declaration of Helsinki. All procedures involved in this study will be approved by the Ethical Committee of the Diabetic Association of Bangladesh for Medical Research. Both witnessed and formally recorded verbal informed consent will be obtained from each subject along with written consent prior to inclusion in the study. This method was used to avoid selection bias due to the high literacy level of Bangladesh. Study participants will also be verbally informed of their right to

withdraw from the study at any stage or to omit their data from the analysis. Data collected for this study will be stored in a way that separated personal identifiers from all samples collected and the responses to all survey questions. Therefore, it will not be possible to identify respondents either directly or through identifiers linked to them. We will seek Clinical Trial Registration.

15. Funding of the study/ budget:

The total cost of the project will be met by the following sources:

- Non-Communicable Disease Control Program of Directorate General of Health Services, Ministry of Health and Family Welfare, Government of the People's Republic of Bangladesh

16. Staff of the study:

One (1) Principal Investigator, two (2) CO-PI, nine (9) Investigators, one (1) statistician, sixteen (16) Field Officers, twenty (20) Volunteers, one (1) Admin and Finance Officer, and one (1) Office Assistant will be recruited to conduct the study.

17. Responsibilities of staff:

Principal investigator (PI): PI, who will be employed for the whole period of study, will be responsible for the following:

- Overall coordination of the project
- Protocol development and ethical committee approval
- Liaising with funding agency
- Data clinging and analysis
- Report writing and dissemination

Co-Investigator (Co-PI): who will be employed for the whole period of study, will be responsible for the following:

- Coordination of project staff and investigators
- Protocol development and ethical committee approval
- Field visits
- Available 24 hours for assistance in case of any adverse events
- Data clinging and analysis

Field Officers: Sixteen (16) physicians working in different BADAS and its AAs will be employed for subject recruitment periods, and will be responsible for the following:

- Conducting structured interviews
- Clinical assessment
- Treatment if needed

Volunteers: The volunteers, who will be employed for subject recruitment periods, will be responsible for the following:

- First contact person who will discuss the study with the participants
- Obtaining informed consent
- Collection of anthropometric data
- Data entry

Admin and finance officer: The admin and finance officer will be employed for 8 months. He will be responsible for the following:

- Administrative and logistical activities
- Day to day finance and accounts related activities
- Financial reporting
- Supporting other staff

Office assistant: The finance assistant will be employed for 8 months.

- Messenger
- Photocopying documents
- Posting letters
- Storing papers, equipment

18. Governance and quality control

18.1 Data Monitoring and Ethics Committee (DMEC):

The **Data Monitoring and Ethics Committee (DMEC)**, comprising Prof. Dr. SM Ashrafuzzaman, Dr. Abdul Alim, Sharif Mahmood, and Prof. Samsad Jahan, will oversee the ethical conduct of the study and monitor data quality and safety. The DMEC will:

- Review study progress and ensure compliance with ethical guidelines.
- Monitor adverse events and ensure participant safety.
- Evaluate data quality and integrity throughout the study.

18.2 Trial Steering Committee (TSC):

The Trial Steering Committee (TSC), consisting of Prof. Akhtar Husain, Prof. AK Azad Khan, Prof. Tofail Ahmed, Arifur Rahman, and Sharif Mahmood, will provide strategic oversight and guidance for the study. The TSC will:

- Review and approve the study protocol and any amendments.
- Monitor overall study progress and ensure alignment with research objectives.
- Provide expert advice on scientific, logistical, and ethical matters.

Role of the Committees:

- Ethical Oversight: The DMEC will ensure that the study adheres to ethical standards and protects participant rights.
- Data Integrity: Both committees will monitor data collection and analysis to ensure accuracy and reliability.

- Study Progress: The TSC will provide guidance on study implementation and address any challenges that arise during the trial.

The study team will acknowledge the invaluable contributions of the Data Monitoring and Ethics Committee and the Trial Steering Committee for their guidance and support in ensuring the study's success. Their expertise and oversight will be critical in maintaining the study's ethical and scientific standards.

18.3. Data handling and record keeping:

The following steps will be followed –

18.3.1. Confidentiality:

Information about study subjects will be kept confidential and managed according to the existing rules of the institutional review committee.

18.3.2. Records retention:

The correlation tool will be destroyed at the completion of the study in accordance with the health documentation destruction policy of the institutional review committee.

18.3.3. Regulatory binder:

A regulatory binder will be maintained for this study. This will include items such as this protocol, the letter of approval from the IRB, the Waiver of the Authorization form, and all other information pertinent to this study.

19. Reporting adverse events (AEs)

Two investigators will be available 24 hours for assistance in case of any adverse events. Subjects will have 24 hours access to emergency inpatient services if related to the study. Records will be kept of any adverse effects occurring during the study and sent to the governance committees.

20. Indemnity

The study will be indemnified by the Diabetic Association of Bangladesh.

21. Future perspectives

Our findings will help to compile large hospital-based data and create an environment that will be conducive to promoting healthy lifestyles through multi-sectorial, inter-disciplinary collaborations.

22. Conflict of interest:

No potential conflict of interest.

23. Report writing of the project:

A report of the project will be delivered monthly by the investigator. A detailed report writing format will be developed based on the objectives of the study. Moreover, reports including financial reports will be submitted to BADAS and the funding agency at the end of the study.

24. Dissemination of the results:

Scientific results will be published in international journals of diabetes or epidemiology. In addition, we will publish summary articles in the Journal of the Bangladesh Medical Association and the Medical Journal of BADAS. Further, results will be presented and discussed among the stakeholders in Bangladesh and at international conferences.

25. Time frame:

The duration of the study will be twelve months.

Description/ Time in 1 months	1	2	3	4	5	6	7	8	9	10	11	12
Finalize the methodology, field assessment												
Staff recruitment and training												
Development of data collection tool												
Ethical approval												
Screening & patient enrollment												
Baseline information collection												
Intervention tool development												
Baseline assessment												
Baseline data analysis												
Intervention												
End line Assessment												
End line and total data analysis												
Report writing												
Dissemination and validation												
Final Report Submission												

26. Budget:

Cost Component	Costs (BDT)
Staff Remuneration	1498720.00
Laboratory Investigations	3,233,720.00
Drafting and Printing	180,000.00
Travel and Subsistence:	1,190,000.00
Program Expenditure	2,998,000.00
Equipments, Instruments etc.	160,000.00
Use of Computer and Software	400,000.00
Other Administrative Expenses	302,400.00

**Grand Total**	**9,962,840.00**

27. References:

1. International Diabetes Federation. Diabetes Atlas. 10th edn. Brussels: International Diabetes Federation, 2021.
2. Akhtar S, Nasir JA, Sarwar A, et al. Prevalence of diabetes and pre-diabetes in Bangladesh: a systematic review and meta-analysis. BMJ Open 2020;10: e036086. doi:10.1136/ bmjopen-2019-036086.
3. Bhowmik B, Binte Munir S, Ara Hossain I, et al. Prevalence of type 2 diabetes and impaired glucose regulation with associated cardiometabolic risk factors and depression in an urbanizing rural community in Bangladesh: a population-based cross-sectional study. Diabetes Metab J 2012;36(6):422-32. doi: 10.4093/dmj.2012.36.6.422. Epub 2012 Dec 12. PMID: 23275936; PMCID: PMC3530713.
4. Bhowmik B, Siddiquee T, Mujumder A, et al. Serum Lipid Profile and Its Association with Diabetes and Prediabetes in a Rural Bangladeshi Population. Int J Environ Res Public Health. 2018; 15:1944. doi: 10.3390/ijerph15091944. PMID: 30200612; PMCID: PMC6165005.
5. Afroz A, Alam K, Hossain N, et al. Burden of macro- and micro-vascular complications of type 2 diabetes in Bangladesh. Diabetes & Metabolic Syndrome: Clinical Research & Reviews 2019;13: 1615-1622.
6. Afroz A, Alam K, Ali L, et al. Type 2 diabetes mellitus in Bangladesh: a prevalence-based cost-of-illness study. BMC Health Serv Res 2019; 19:1–12.doi:10.1186/s12913-019-4440-3.
7. Lewis CP, Newell JN. Patients' perspectives of care for type 2 diabetes in Bangladesh -a qualitative study. BMC Public Health. 2014; 14:737. doi: 10.1186/1471-2458-14-737.
8. Spijkerman AMW, Henry RMA, Dekker JM, et al. Prevalence of macrovascular disease amongst type 2 diabetic patients detected by targeted screening and patients newly diagnosed in general practice: the Hoorn Screening Study. J Intern Med 2004; 256: 429–436.
9. Spijkerman AMW, Dekker JM, Nijpels G, et al. Microvascular complications at time of diagnosis of type 2 diabetes are similar among diabetic patients detected by targeted screening and patients newly diagnosed in general practice. Diabetes Care 2003; 26: 2604–2608.
10. Haffner SM, Stern MP, Hazuda HP, et al. Cardiovascular risk factors in confirmed prediabetic individuals. Does the clock for coronary heart disease start ticking before the onset of clinical diabetes? JAMA 1990; 263: 2893–2898.
11. Tuomilehto J. Nonpharmacologic therapy and exercise in the prevention of type 2 diabetes. Diabetes Care 2009; 32 (Suppl 2): 189 – 93.
12. Hjellset VT. A culturally adapted lifestyle intervention with main focus on blood glucose regulation improved the risk profi le for type 2 diabtes in pakistani immigrant women: they are not aliens. Faculty of Medicine, University of Oslo; 2011.
13. Sakane N, Sato J, Tsushita K, Tsujii S, Kotani K, Tsuzaki K, et al. Prevention of type 2 diabetes in a primary healthcare setting: three-year results of lifestyle intervention in Japanese subjects with impaired glucose tolerance. BMC Public Health 2011; 11 (1): 40.
14. Diabetes Prevention Program Research Group. Reduction in the incidence of type 2 diabetes with lifestyle intervention or metformin. N Engl J Med 2002; 346: 393–403.
15. Ramachandran A, Snehalatha C, Mary S, et al. The Indian Diabetes Prevention Programme shows that lifestyle modification and metformin prevent T2DM in Asian Indian subjects with impaired glucose tolerance (IDPP-1). Diabetologia 2006; 49: 289–297.
16. https://cghr-badas.org/imam/ (last access January 2021).
17. Hills AP, Arena R, Khunti K, et al. Epidemiology and determinants of type 2 diabetes in south Asia. Lancet Diabetes Endocrinol. 2018;6(12):966-978. doi: 10.1016/S2213-8587(18)30204-3.

18. Misra A, Sattar N, Tandon N, et al. Clinical management of type 2 diabetes in south Asia. Lancet Diabetes Endocrinol. 2018;6(12):979-991. doi: 10.1016/S2213-8587(18)30199-2.
19. Hills AP, Misra A, Gill JMR, et al. Public health and health systems: implications for the prevention and management of type 2 diabetes in south Asia. Lancet Diabetes Endocrinol. 2018;6(12):992-1002. doi: 10.1016/S2213-8587(18)30203-1.
20. Sarker AR, Sultana M. Health and economic burden of diabetes in Bangladesh: Priorities for attention and control. J Diabetes. 2017 Dec;9(12):1118-1119. doi: 10.1111/1753-0407.12587. Epub 2017 Sep 13. PMID: 28749030.
21. https://pcc-badas.org/ (last access January 2021.
22. Fottrell E, Ahmed N, Morrison J, et al. Community groups or mobile phone messaging to prevent and control type 2 diabetes and intermediate hyperglycemia in Bangladesh (DMagic): a cluster-randomised controlled trial. Lancet Diabetes Endocrinol. 2019;7(3):200-212. doi: 10.1016/S2213-8587(19)30001-4.
23. Sattin RW, Williams LB, Dias J, Garvin JT, Marion L, Joshua TV, et al. Community trial of a faith-based lifestyle intervention to prevent diabetes among African-Americans. J Comm Health 2016;41(1):87–96.
24. Williams LB, Sattin RW, Dias J, Garvin JT, Marion L, Joshua T, et al. Design of a cluster-randomized controlled trial of a diabetes prevention program within African-American churches: the Fit Body and Soul study. Contemp Clin Trials 2013;34(2):336–47.
25. Resnicow K, Kramish Campbell M, Carr C, McCarty F, Wang T, Periasamy S, et al. Body and soul: A dietary intervention conducted through African-American churches. Am J Prev Med. 2004; 27:97–105. [PubMed: 15261895]
26. Stokols D. Translating social ecological theory into guidelines for community health promotion. Am J Health Promot. 1996; 10:282–298. [PubMed: 10159709]
27. Dodani S, Fields JZ. Implementation of the Fit Body and Soul, a church-based life style program for diabetes prevention in high-risk African Americans. The Diabetes Educ. 2010; 36:465–472.
28. Cohen-Dar, M., Obeid, S. Islamic Religious Leaders in Israel as Social Agents for Change on Health-Related Issues. J Relig Health 56, 2285–2296 (2017). https://doi.org/10.1007/s10943-017-0409-x
29. Allison Ruark, Jane Kishoyian, Mona Bormet, Douglas Huber. Increasing Family Planning Access in Kenya Through Engagement of Faith-Based Health Facilities, Religious Leaders, and Community Health Volunteers. Global Health: Science and Practice Sep 2019, 7 (3) 478-490; DOI: 10.9745/GHSP-D-19-00107.
30. UNFPA Bangladesh. 2022 State of the World Population Report. Available from: https://bangladesh.unfpa.org/en/news/2022-state-world-population-report.
31. PopulationPyramid.net. Bangladesh Population 2022. Available from: https://www.populationpyramid.net/bangladesh/2022/.
32. Bhowmik B, Akhter A, Ali L, Ahmed T, Pathan F, Mahtab H, Khan AK, Hussain A. Simple risk score to detect rural Asian Indian (Bangladeshi) adults at high risk for type 2 diabetes. J Diabetes Investig. 2015; 6:670-7. doi: 10.1111/jdi.12344.
33. World Health Organization. Definition and Diagnosis of Diabetes Mellitus and Intermediate Hyperglycemia: Report of a WHO/IDF Consultation. Geneva, World Health Org., 2006.
34. Asghar S, Khan AK, Ali SM, Sayeed MA, Bhowmik B, Diep ML, Shi Z, Hussain A. Incidence of diabetes in Asian-Indian subjects: a five year follow-up study from Bangladesh. Prim Care Diabetes. 2011 Jul;5(2):117-24. doi: 10.1016/j.pcd.2011.01.002. Epub 2011 Feb 9. PMID: 21306967.
35. Choo V. WHO reassesses appropriate body-mass index for Asian populations. Lancet 2002; 360: 235.
36. World Health Organization, Western Pacific Region. The International Association for the Study of Obesity and the International Obesity Task Force. The Asia–Pacific Perspective: Redefining Obesity and its

Treatment. Health Communications Australia Pty Limited, Sydney, Australia, 2000. Available at: www.diabetes.com.au/pdf/obesity_report.pdf (Last accessed May 23 2021).
37. Guidelines Subcommittee. 1999 World Health Organization-International Society of Hypertension guidelines for the management of hypertension. J Hypertens 1999; 17:151—183.
38. Expert Panel on Detection E, and Treatment of High Blood Cholesterol in Adults. Executive Summary of The Third Report of The National Cholesterol Education Program (NCEP) Expert Panel on Detection, Evaluation, And Treatment of High Blood Cholesterol In Adults (Adult Treatment Panel III). JAMA 2001; 285: 2486-97.
39. Khan MMH, Aklimunnessa K, Kabir MA, et al. Determinants of physical activity among urban adults in Bangladesh: a case-control study. J Phys Act Health 2006; 3(4): 424–38. DOI: 10.1123/jpah.3.4.424.
40. Zaman MM, Bhuiyan MR, Karim MN, et al. Physical activity levels and associated factors among adults in rural Bangladesh: a cross-sectional study. BMC Public Health 2019; 19: 1467. DOI: 10.1186/s12889-019-7795-9.
41. Islam FMA, Chakrabarti R, Dirani M, et al. Knowledge, attitudes, and practice of diabetes in rural Bangladesh: the Bangladesh Population-based Diabetes and Eye Study (BPDES). *PLoS One* 2014; 9(10): e110368. DOI: 10.1371/journal.pone.0110368
42. Balestroni G, Bertolotti G. an instrument for measuring quality of life. Monaldi Arch Chest Dis. 2012;78(3):155-9. Italian. doi: 10.4081/monaldi.2012.121. PMID: 23614330.

Statistical Analysis Plan (SAP)

Study Title: Faith-Based Lifestyle Intervention to Prevent Type 2 Diabetes Mellitus in Rural Bangladesh: A Cluster-Randomized Clinical Trial
Trial Registration: ISRCTN91564707
SAP Version: Retrospective Version 1.0 **Date:** 20 August 2023

Declaration: This SAP was developed retrospectively after completion of data collection and before analysis of primary outcomes. It reflects the prespecified analytical approach used in the manuscript. The SAP adheres to CONSORT 2010 guidelines for cluster-randomized trials.

1. Study Overview

This cluster-randomized controlled trial (cRCT) evaluated the effectiveness of a culturally tailored, mosque-based lifestyle intervention in preventing type 2 diabetes mellitus (T2DM) among adults with prediabetes in rural Bangladesh. Eight mosques were randomized (1:1) to intervention or control arms using a computer-generated block randomization schedule (block size = 2), stratified by district. Allocation was performed by an independent statistician; field staff enrolling participants were unaware of allocation until post-randomization. The study followed participants over 12 months.

Intervention: Monthly sermons by imams and trained female assistants (promoted healthy dietary practices, physical activity, and diabetes awareness. Control mosques received standard health education materials without structured sessions.

Blinding: Outcome assessors were blinded to allocation, but participants and intervention deliverers were not due to the behavioral nature of the trial.

2. Objectives and Hypotheses

Primary Objective: To determine whether the intervention reduced the cumulative 12-month incidence of T2DM among individuals with prediabetes.

Secondary Objectives: To assess the intervention's effect on body weight, BMI, glycemic indices (FPG, 2hPG, HbA1c), lipid profiles, physical activity, diabetes knowledge, and quality of life.

Hypothesis: The intervention group had a lower incidence of T2DM and greater improvements in cardiometabolic outcomes than the control group.

3. Trial Design and Randomization

A cluster-randomized parallel-group design was used with 8 mosques as units of randomization. Mosques were randomized 1:1 to intervention or control groups using a computer-generated block randomization schedule (block size = 2), stratified by district. The allocation sequence was prepared by an independent statistician. Field staff enrolled participants and assigned them post-randomization.

4. Sample Size

Cluster sizes were assumed to be equal in sample size calculation; actual variability was minor (range 92–110).
Calculated to detect a 7% absolute reduction in diabetes incidence (control assumed 12% incidence), with 80% power, 5% significance, and design effect (ICC=0.041 from pilot data).
Final sample: 799 participants (396 intervention, 403 control) after exclusions (25 excluded due to [specify reasons: e.g., baseline diabetes, withdrawal]).
Achieved power: 82% post-hoc (given observed ICC=0.039).

5. Analysis Populations

- **Intention-to-Treat (ITT):** All randomized participants analyzed per original allocation.
- **Modified ITT:** Excludes only participants with no post-baseline data (n=10).
- **Per-Protocol (PP):** Intervention group restricted to participants with ≥75% attendance (verified by mosque sign-in sheets).

6. Handling of Missing Data

Primary: Complete-case analysis. Sensitivity analyses:

- **Multiple imputation (MI):** 10 imputations using chained equations (predictors: age, sex, baseline HbA1c, mosque cluster).
- **Best-case/Worst-case scenarios:** Best-case assumes dropouts had no diabetes; worst-case assumes all developed diabetes.
- **Pattern-mixture models** to test missing-not-at-random (MNAR) assumptions.

Report: Flow diagram with missing data by arm and reasons.

No formal adjustment for multiplicity was applied; secondary outcome results should be interpreted as exploratory.

7. Statistical Methods

7.1 Baseline Comparisons

- Baseline characteristics were summarized as means (SDs) or proportions.
- Group comparisons used independent t-tests (continuous) and chi-square tests (categorical).
- Formal hypothesis testing of baseline differences was descriptive only.

7.2 Primary Outcome

Outcome: Cumulative incidence of T2DM at 12 months, diagnosed using WHO 2006 criteria.

Analysis:
- Kaplan-Meier survival curves estimated cumulative incidence.
- Group comparisons were assessed via log-rank test.
- Cox proportional hazards model with shared frailty for mosque-level clustering was used.
- HRs with 95% CIs were reported.
- The assumption of proportional hazards was tested using Schoenfeld residuals.

7.3 Secondary Outcomes

Outcomes: Weight, BMI, FPG, 2hPG, HbA1c, HDL, blood pressure, physical activity, diabetes knowledge, EQ-5D-5L score.

Analysis:
- Linear mixed-effects models were used for continuous outcomes with mosque-level random intercepts.
- Generalized estimating equations (GEE) were used for binary outcomes with robust SEs and exchangeable correlation structure.
- All models adjusted for baseline values.
- Difference-in-differences approach was used to estimate net intervention effect.

8. Subgroup and Sensitivity Analyses

- Subgroup analyses were conducted by age group (<45 vs ≥45 years) and sex.
- Sensitivity analyses used imputed datasets.
- Dropout prediction performance was evaluated using AUC, sensitivity, specificity, F1 score.
- ICC was calculated for the primary outcome.

9. Statistical Software

All analyses were performed using STATA version 17.0 (StataCorp, College Station, TX, USA). MI via 'mi estimate', GEE via 'xtgee'. A two-sided p-value <0.05 was considered statistically significant.

10. Deviations from the Protocol

- No changes were made to the primary or secondary outcome definitions.
- Per-protocol and imputation-based sensitivity analyses were added during analysis to enhance robustness.

11. Trial Reporting

This SAP was written in alignment with the CONSORT 2010 statement and its extension for cluster-randomized trials. Results were reported in accordance with JAMA Network Open's statistical reporting standards.
