# Supplementary material for: Faith-Based Lifestyle Intervention for Diabetes Prevention Among Adults in Bangladesh: A Cluster Randomized Clinical Trial
Source: JAMA Netw Open. 2025 Oct 20;8(10):e2538101. doi: 10.1001/jamanetworkopen.2025.38101 (PMC12538358; doi:10.1001/jamanetworkopen.2025.38101)
Supplement: Supplement 2. — eTable 1. Baseline Characteristics of Participants by Mosque Cluster eTable 2. Baseline Characteristics of Participants Who Completed 12-Month Follow-Up vs Those Lost to Follow-Up eTable 3. Primary and Secondary Outcomes at 12 Months: Intervention vs Control eTable 4. Sensitivity Analyses of Dropout Prediction Model eTable 5. Model Fit and Diagnostics [file jamanetwopen-e2538101-s002.pdf]

Supplemental Online Content

Bhowmik B, Siddiquee T, Munir SB, et al. Faith-based lifestyle intervention for diabetes prevention among adults in Bangladesh: a cluster randomized clinical trial. *JAMA Netw Open*. 2025;8(10):e2538101. doi:110.1001/jamanetworkopen.2025.38101

eTable 1. Baseline Characteristics of Participants by Mosque Cluster

eTable 2. Baseline Characteristics of Participants Who Completed 12-Month Follow-Up vs Those Lost to Follow-Up

eTable 3. Primary and Secondary Outcomes at 12 Months: Intervention vs Control

eTable 4. Sensitivity Analyses of Dropout Prediction Model

eTable 5. Model Fit and Diagnostics

This supplemental material has been provided by the authors to give readers additional information about their work.

© 2025 Bhowmik B et al. *JAMA Network Open*.

eTable 1. Baseline Characteristics of Participants by Mosque Cluster

Variable	M-1	M-2	M-3	M-4	M-5	M-6	M-7	M-8
Participant	103	102	99	103	97	98	**94**	**103**
Age (years)	45.1 (11.7)	46.3 (12.9)	45.6 (12.8)	46.3 (12.5)	48.9 (11.3)	46.3 (11.0)	44.5 (10.3)	45.5 (9.8)
Sex (Male)	49.5 (51)	43.1 (44)	48.5 (48)	42.7 (44)	55.7 (54)	45.9 (45)	39.4 (37)	50.5 (52)
Weight (kg)	65.3 (12.0)	62.1 (11.2)	61.9 (12.2)	62.8 (12.3)	62.0 (10.3)	65.1 (12.6)	62.1 (12.8)	65.2 (11.0)
BMI (kg/m2)	26.5 (4.3)	25.4 (3.8)	25.4 (4.1)	26.5 (4.7)	25.3 (3.9)	26.6 (4.4)	26.4 (4.5)	25.7 (3.9)
WC (cm)	90.3 (9.3)	91.2 (10.3)	90.3 (12.0)	89.6 (12.3)	89.1 (15.3)	91.1 (13.9)	86.2 (10.1)	87.9 (10.5)
SBP (mmHg)	134.9 (19.9)	129.1 (18.0)	129.0 (19.9)	128.5 (20.1)	127.3 (19.1)	126.0 (17.4)	128.4 (20.5)	129.6 (20.3)
DBP (mmHg)	82.8 (9.8)	79.9 (10.3)	79.3 (10.4)	80.9 (11.7)	78.9 (10.5)	78.5 (10.6)	81.1 (11.8)	82.2 (12.4)
FBG (mmol/L)	6.0 (0.48)	6.2 (0.35)	6.2 (0.39)	6.1 (0.50)	6.1 (0.44)	6.1 (0.48)	6.1 (0.46)	6.2 (0.47)
2hBG (mmol/L)	8.2 (1.1)	7.9 (1.2)	8.0 (1.2)	8.1 (1.3)	8.2 (1.3)	8.2 (1.4)	8.0 (1.4)	8.0 (1.6)
HbA1c (%)	5.9 (0.43)	5.9 (0.36)	5.8 (0.30)	5.9 (0.44)	5.8 (0.40)	5.8 (0.30)	5.9 (0.41)	5.8 (0.46)
T-Chol (mg/dl)	187.2 (45.5)	174.6 (42.6)	184.4 (45.9)	183.9 (40.8)	186.1 (47.9)	183.9 (40.4)	177.2 (40.1)	179.8 (43.1)
Tg (mg/dl)	214.2 (119.0)	182.8 (107.6)	198.4 (122.0)	197.7 (113.7)	196.7 (113.7)	211.9 (116.6)	187.5 (114.8)	186.6 (96.1)
HDL-C (mg/dl)	37.5 (11.7)	38.7 (11.5)	36.0 (10.4)	37.5 (10.2)	38.5 (11.2)	36.8 (10.5)	37.6 (10.5)	37.9 (11.1)
HDL-C (mg/dl)	115.1 (35.4)	105.2 (31.7)	118.9 (29.3)	112.1 (33.1)	116.9 (40.8)	110.8 (31.7)	112.4 (33.2)	112.5 (33.3)

Data are presented as a mean (± SD) for continuous variables and % (number) for categorical variables. Abbreviation: M-Mosque; M-1: Keranigan Upazila; M-2: Shibaloy Upazila; M-3: Monohardi Upazila, M-4: Raipura Upazila; M-5: Dhanbari Upazila, M-6: Singair Upazila; M-7: Sreenagar Upazila; M-8: Sakhipur Upazil;, BMI, body mass index; WC, waist circumference; SBP, systolic blood pressure; DBP, diastolic blood pressure; FBG, fasting blood glucose; 2hBG, 2 hours after blood glucose; HbA1c, glycated hemoglobin; T-Chol, total cholesterol; Tg, triglycerides; HDL-C, high density lipoprotein cholesterol; LDL-C, low density lipoprotein cholesterol.

© 2025 Bhowmik B et al. *JAMA Network Open*.

eTable 2. Baseline Characteristics of Participants Who Completed 12-Month Follow-Up vs Those Lost to Follow-Up

Variable	Completers	Lost to Follow-up	Group-Difference	P value
Number	**641**	**158**		
Age	46.1 (11.5)	46.3 (12.2)	−0.2 (−2.2, 1.9)	0.862
Sex, Female, % (n)	54.5 (351)	47.1 (73)	+7.4 (−1.6, 16.4)	0.097
Physical activity, % (n)	42.2 (272)	46.5 (72)	−4.3 (−12.9, 4.3)	0.341
DM Knowledge (some), % (n)	39.1 (248)	36.3 (53)	+2.8 (−6.3, 11.9)	0.529
Weight (kg)	60.8 (12.0)	62.2 (12.3)	−1.5 (−3.6, 0.7)	0.181
BMI (kg/m^2)	25.1 (4.1)	25.0 (4.6)	+0.1 (−0.7, 0.8)	0.954
WC (cm)	88.3 (12.0)	88.2 (12.0)	+0.2 (−1.9, 2.2)	0.889
SBP (mmHg)	127.3 (19.7)	129.4 (19.6)	−2.1 (−5.5, 1.4)	0.242
DBP (mmHg)	80.8 (11.3)	81.9 (10.8)	−1.1 (−3.1, 0.8)	0.255
FBG (mmol/L)	6.1 (0.5)	6.1 (0.5)	−0.1 (−0.1, 0.0)	0.235
2hBG (mmol/L)	7.8 (1.3)	7.6 (1.4)	+0.2 (0.0, 0.5)	0.065
HbA1c (%)	5.8 (0.38)	5.9 (0.54)	−0.07 (−0.19, 0.04)	0.214
T-Chol (mg/dl)	182.0 (42.7)	182.7 (46.4)	−0.8 (−8.6, 7.1)	0.849
Tg (mg/dl)	192.0 (110.6)	199.6 (124.2)	−7.6 (−28.1, 12.8)	0.464
HDL-C (mg/dl)	37.0 (11.2)	37.3 (9.4)	−0.3 (−2.3, 1.7)	0.751
LDL-C (mg/dl)	112.5 (32.9)	112.2 (35.3)	+0.2 (−6.1, 6.6)	0.942

Data are presented as a mean (± SD) for continuous variables and % (number) for categorical variables. Group differences for continuous variables are mean differences (95% CI); for categorical variables, they are percentage point differences (95% CI). p values from independent t-tests (continuous) or χ^2 tests (categorical). Abbreviation: DM, diabetes mellitus; BMI, body mass index; WC, waist circumference; SBP, systolic blood pressure; DBP, diastolic blood pressure; FBG, fasting blood glucose; 2hBG, 2 hours after blood glucose; HbA1c, glycated hemoglobin; T-Chol, total cholesterol; Tg, triglycerides; HDL-C, high density lipoprotein cholesterol; LDL-C, low density lipoprotein cholesterol.

© 2025 Bhowmik B et al. *JAMA Network Open*.

eTable 3. Primary and Secondary Outcomes at 12 Months: Intervention vs Control

Outcome metric	Control group (n=403)	Intervention group (n=396)	Between-group difference (95% CI)	P-value
**12-month cumulative incidence, % (n)**	17.1 (69)	9.8 (39)	**−7.3%** (−11.9 to −2.6)	0.002
**Incidence rate (per 1,000 person-years)**	16.4 (12.5–20.3)	8.9 (6.1–11.7)	**−7.5** (−12.1 to −2.9)	0.002
**Absolute risk reduction (ARR), %**	—	—	7.3 (5.22–10.6)	0.002
**Relative risk reduction (RRR), %**	—	—	43 (15–70)	0.002
**Number needed to treat (NNT)**	—	—	14 (9–39)	0.002
**Hazard ratio (95% CI)**	Reference	0.75 (0.60–0.95)	—	0.02

© 2025 Bhowmik B et al. *JAMA Network Open*.

eTable 4. Sensitivity Analyses of Dropout Prediction Model

**Analysis Dimension**	**Methodological Approach**	**Primary Outcome (AUC) [95% CI]**	**Secondary Outcomes**	**Key Clinical Interpretation**
Missing Data Handling	Complete-case analysis (n=572)	0.712 [0.673–0.751]	Sens: 0.647 [0.591–0.703]; Spec: 0.761 [0.725–0.797]	Limited generalizability due to exclusion of 28% cases
	Multiple Imputation (MICE, m=5)	0.728 [0.690–0.766]	Sens: 0.683 [0.628–0.738]; Spec: 0.774 [0.739–0.809]	Preferred approach preserves sample size and power
Subgroup Analyses	Age <45 years (n=387)	0.701 [0.653–0.749]	PPV: 0.412 [0.351–0.473]; NPV: 0.892 [0.863–0.921]	Model performs better in older participants
	Age ≥45 years (n=412)	0.745 [0.698–0.792]	PPV: 0.538 [0.471–0.605]; NPV: 0.914 [0.888–0.940]	
	Female participants (n=448)	0.735 [0.689–0.781]	F1: 0.575 [0.519–0.631]; Brier: 0.172	Sex-specific models show comparable performance
	Male participants (n=351)	0.719 [0.666–0.772]	F1: 0.543 [0.481–0.605]; Brier: 0.179	
Threshold Analysis	Default (0.5)	0.728 [0.690–0.766]	Sens: 0.683; Spec: 0.774	Balanced approach
	Clinical Optimal (0.37)	0.728 [0.690–0.766]	Sens: 0.752; Spec: 0.718	Better for early intervention
	High Specificity (0.6)	0.728 [0.690–0.766]	Sens: 0.529; Spec: 0.843	Better for resource allocation
Robustness Checks	Bootstrap Validation (1000 reps)	0.725 [0.702–0.748]	ICC: 0.941 [0.927–0.955]	Model shows high internal consistency
	Time-to-Event Analysis	C-statistic: 0.738 [0.706–0.770]	HbA1c HR: 1.41 [1.22–1.63]	Confirms predictive value of glycemic measures

Abbreviation: AUC, Area Under the Curve; Sen, Sensitivity; MICE, Multiple Imputation by Chained Equations; PPV, Positive Predictive Value; F1, F1 Score; ICC, Intraclass Correlation Coefficient; HbA1c, Glycated Haemoglobin.

© 2025 Bhowmik B et al. *JAMA Network Open*.

eTable 5. Model Fit and Diagnostics

Model Type	Fit Statistics	Diagnostics Performed	Findings / Interpretation
Cox Proportional Hazards (Primary Outcome)	AIC: 2453.1<br>Log-likelihood: -1217.6	Schoenfeld residuals<br>Log-minus-log survival plots	No violation of proportional hazards assumption
Linear Mixed-Effects Model (Continuous Outcomes)	AIC: 3130.4<br>BIC: 3198.2	Residual plot<br>Normality of residuals<br>Random effects variance	Model fit adequate; residuals normally distributed
Generalized Estimating Equations (Binary Outcomes)	QIC: 2287.5<br>Correlation structure: Exchangeable	Wald tests<br>Robust standard errors<br>Covariance matrix inspection	Model converged; estimates robust to correlation structure
Poisson Regression (Sensitivity Analysis)	Deviance: 456.7<br>Pearson χ^2: 478.2	Overdispersion test<br>Residual deviance per df	No significant overdispersion detected
Interval-Censored Cox Model	Log-likelihood: -1195.3<br>AIC: 2402.6	Visual inspection of interval bounds<br>Goodness-of-fit plots	Consistent results with primary Cox model
Discrete-Time Survival (Cloglog Model)	AIC: 2480.2<br>Pseudo R^2: 0.114	Link function check<br>Time interval adequacy	Model appropriate for discrete assessments

Abbreviation: AIC, Akaike Information Criterion; BIC, Bayesian Information Criterion; QIC, Quasi-likelihood under the Independence model Criterion; df, degree of freedom.

© 2025 Bhowmik B et al. *JAMA Network Open*.
