# Supplementary material for: Faith-Based Lifestyle Intervention for Diabetes Prevention Among Adults in Bangladesh: A Cluster Randomized Clinical Trial
Source: JAMA Netw Open. 2025 Oct 20;8(10):e2538101. doi: 10.1001/jamanetworkopen.2025.38101 (PMC12538358; doi:10.1001/jamanetworkopen.2025.38101)
Supplement: Supplement 3. — Data Sharing Statement [file jamanetwopen-e2538101-s003.pdf]

Data Sharing Statement

Bhowmik. Faith-Based Lifestyle Intervention for Diabetes Prevention Among Adults in Bangladesh. *JAMA Netw Open*. Published October 20, 2025.
doi:10.1001/jamanetworkopen.2025.38101

Data
Additional Information: ISRCTN-ISRCTN91564707
Data available: Yes
Data types: Deidentified participant data
How to access data: Requests for data access should be directed to the corresponding author, Prof. Bishwajit Bhowmik, via email at cghr@dab-bd.org.
When available: beginning date: 12-08-2025, end date: 12-08-2027

Supporting Documents
Document types: None

Additional Information
Who can access the data: Data will be made available to researchers whose proposed use of the data has been approved by the corresponding author.
Types of analyses: Data will be made available for any purpose, upon approval.
Mechanisms of data availability: Data will be shared after approval of a proposal and with a signed data access agreement.
Any additional restrictions: None
